# Engineered live bacteria suppress *Pseudomonas aeruginosa* infection in mouse lung and dissolve endotracheal-tube biofilms

**DOI:** 10.1038/s41587-022-01584-9

**Published:** 2023-01-19

**Authors:** Rocco Mazzolini, Irene Rodríguez-Arce, Laia Fernández-Barat, Carlos Piñero-Lambea, Victoria Garrido, Agustín Rebollada-Merino, Anna Motos, Antoni Torres, Maria Jesús Grilló, Luis Serrano, Maria Lluch-Senar

**Affiliations:** 1 Centre for Genomic Regulation, Barcelona Institute of Science and Technology, Barcelona, Spain; 2 Pulmobiotics Ltd, Barcelona, Spain; 3 Institute of Agrobiotechnology, CSIC-Navarra Government, Navarra, Spain; 4 Cellex Laboratory, CibeRes, Institut d’Investigacions Biomèdiques August Pi i Sunyer, University of Barcelona, Barcelona, Spain; 5 Department of Pneumology, Thorax Institute, Hospital Clinic of Barcelona, SpainICREA, Barcelona, Spain; 6 VISAVET Health Surveillance Centre, Complutense University of Madrid, Madrid, Spain; 7 Department of Internal Medicine and Animal Surgery, Faculty of Veterinary Medicine, Complutense University of Madrid, Madrid, Spain; 8 Universitat Pompeu Fabra, Barcelona, Spain; 9 ICREA, Barcelona, Spain; 10 Basic Sciences Department, Faculty of Medicine and Health Sciences, Universitat Internacional de Catalunya, Sant Cugat del Vallès, Spain

**Keywords:** Synthetic biology, Biotechnology

## Abstract

Engineered live bacteria could provide a new modality for treating lung infections, a major cause of mortality worldwide. In the present study, we engineered a genome-reduced human lung bacterium, *Mycoplasma pneumoniae*, to treat ventilator-associated pneumonia, a disease with high hospital mortality when associated with *Pseudomonas aeruginosa* biofilms. After validating the biosafety of an attenuated *M. pneumoniae* chassis in mice, we introduced four transgenes into the chromosome by transposition to implement bactericidal and biofilm degradation activities. We show that this engineered strain has high efficacy against an acute *P. aeruginosa* lung infection in a mouse model. In addition, we demonstrated that the engineered strain could dissolve biofilms formed in endotracheal tubes of patients with ventilator-associated pneumonia and be combined with antibiotics targeting the peptidoglycan layer to increase efficacy against Gram-positive and Gram-negative bacteria. We expect our *M. pneumoniae*-engineered strain to be able to treat biofilm-associated infections in the respiratory tract.

## Abstract (web summary)

A bacterium expressing antibacterial genes treats a lung infection associated with mechanical ventilators.

## Main

Respiratory diseases are among the top ten causes of death worldwide. Efforts to develop new therapeutics against respiratory tract infections are growing, especially given the mounting concern about antibiotic-resistant bacteria and the paucity of new antibiotics^1^. Moreover, antibiotic therapies eliminate beneficial lung microbes and can lead to the persistence of pathogenic, resistant strains^2^. Approximately 65–80% of human infections are associated with biofilms^3–6^. They are especially frequent in pulmonary chronic diseases^7,8^, including cystic fibrosis (CF)^9^, chronic obstructive pulmonary disease^8^ and non-CF bronchiectasis, as well as in acute airway infections, such as ventilator-associated tracheobronchitis (VAT) and ventilator-associated pneumonia (VAP)^10,11^. Biofilms are an ancestral bacterial survival strategy against environmental stress that consists of complex and dynamic structures formed by aggregates of microorganisms embedded in a polymeric matrix, which confers tolerance to antimicrobials^12,13^ and allows them to evade host defense mechanisms^14^. Thus, biofilms can cause recurrent, device-associated chronic infections. As a result, the effective minimum bactericidal concentrations of antibiotics for biofilm eradication in vivo are relatively high and can cause adverse effects, such as renal and/or hepatic injury^15^. Moreover, many of the lung pathogenic bacterial strains are resistant to antibiotics.

Biofilm formation is especially problematic with the use of endotracheal tubes (ETTs) in patients who require invasive mechanical ventilation (MV) in intensive care units (ICUs). VAT and VAP are estimated to occur in 9–27% of all patients receiving MV^16^. For patients with severe acute respiratory syndrome coronavirus 2 (SARS-CoV-2) who receive MV, the incidence rates of VAP/VAT are higher than usual and exceed 50% overall^17^. Moreover, the mortality rate in VAP patients with COVID-19 was higher than in those with influenza or without viral infection^18,19^ and even higher when associated with *P. aeruginosa* biofilms^20,21^. Until now, most interventions aimed at reducing lung biofilms, including aerosolized antibiotics, have failed or require further investigation^22^.

Engineered bacteria that are genetically modified to treat diseases—which are classified as live biotherapeutic products—could offer effective therapeutic formulations with fewer adverse effects^23–34^. Most bacterial vectors have been designed to treat gut diseases, with a few targeting diseases affecting other organs. Examples include an engineered strain of *Lactobacillus reuteri* that decreases high blood levels of phenylalanine in a homozygous *PAHenu2* (phenylketonuria) mouse model^25^ and an engineered strain of *Lactobacillus* spp. or *Saccharomyces cerevisiae* designed to immunize against HIV in the gut^27^ and the cervicovaginal mucosa^35,36^. However, no bacterial chassis has so far been described for the treatment of lung diseases^37^.

The site of action of a bacterial therapeutic affects the choice of species^38^. Ideally, the selected bacterium should be naturally present in the organ to be treated, to ensure its survival and limit spreading to other organs. For example, the *Escherichia coli* Nissle 1917 strain was engineered to treat *P. aeruginosa* infections in the gut^39^, yet it cannot be used to treat respiratory infections because the respiratory tract is not its natural niche. *M. pneumoniae* is the causative agent of atypical pneumonia and other extrapulmonary pathologies in humans. Compared with other bacterial chassis, *M. pneumoniae* has the following advantages for treatment of lung infections: (1) it has a small genome (816 kbp); (2) it is a mild pathogen that can be eliminated with available antibiotics; (3) it is a bacterium for which more quantitative and extensive datasets are available^40–45^; (4) it has reduced metabolic and genetic networks^46^, which reduce the risk of unwanted interference of the engineered circuits; (5) as it lacks a cell wall, it does not trigger a strong inflammatory response and can be combined with antibiotics that attack the peptidoglycan layer present in cell walls of pathogens (as shown in the present study); (6) its main antigens and virulence factors are well characterized^44,47–49^; (7) genetic tools are available to engineer its genome and to obtain an attenuated strain^50–52^; (8) some *M. pneumoniae* strains, including M129, have a negligible rate of recombination, thereby reducing the risk of horizontal transfer; (9) its UGA codon encodes for tryptophan instead of a translation stop, providing an intrinsic biocontainment mechanism; and (10) it can be grown in a defined, synthetic, serum-free medium to upscale its good manufacturing practice-compliant production^53^.

We used an engineered, attenuated version of *M. pneumoniae* M129 strain as a bacterial chassis to treat/prevent infectious lung diseases caused by *P. aeruginosa*. We first characterized in vivo the safety of use and survival of different attenuated *M. pneumoniae* M129 strains in mouse lungs, to define the optimal chassis. We then engineered this nonpathogenic chassis by introducing two optimized genetic systems: one that combines biofilm dispersal activities (the glycoside hydrolases PelAh^54^ and PslGh^55^ and the A1-II′ alginate lyase^56^) and another that implements antimicrobial activity (pyocin L1 (ref. ^57^) or pyocin S5 (ref. ^58^)) against biofilms formed by *P. aeruginosa*. We validated the activity of the engineered chassis strain in vitro, ex vivo and in vivo. We showed that the engineered strain can reduce an acute *P. aeruginosa* infection in the murine model, thereby improving mouse survival, dissolve biofilms formed in vivo on ETTs in patients with VAP and be combined with antibiotics targeting the bacterial cell wall. Thus, it represents a promising alternative modality for preventing or treating biofilm-associated diseases and eradicating bacterial antibiotic resistance.

## Results

### Delivery and clearance of *M. pneumoniae* strains in murine lung

**Extended Data Fig. 1:**
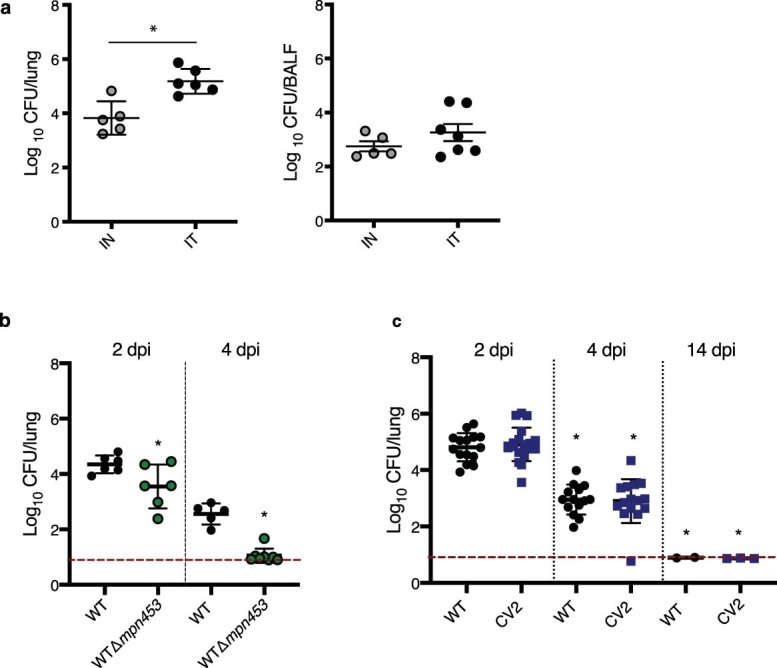
Delivery route and lung infection dynamic of *M. pneumoniae* WT and mutant strains generated in this study. a, CFUs per lung (left) or BALF (right) in CD1 mice infected using the same dose (~1× 10^7^ CFU/mice), by intranasal (IN, 20 µl. Five animals) or intratracheal (IT, 100 µl. Six animals) administration. Mice were euthanized at 2 days post-infection (dpi) to determine bacterial load in lungs (left panel, log_10_ CFU/lung) and in BALF (right panel, log_10_ CFU/lung or BALF). Data are expressed as mean ± standard deviation (SD). Statistical comparison was performed using two-sided t-test. *P = 0.032. b, The WTΔ*mpn453* mutant strain is eliminated faster than the WT strain. Two-sided *t*-test was used (*P = 0.04 and *P* = 0.02). Data are expressed as mean ± standard deviation (SD). c, Lung infection dynamics of *M*. *pneumoniae* WT and CV2 chassis strains. Bacterial counts were obtained at 2 dpi (n = 15), 4 dpi (n = 15) or 14 dpi (n = 3) for both strains. Data are shown as means values ± SD of log_10_ CFU/lung. Statistical comparisons were performed using two-sided *t*-test. *P < 0.05. Red line, experimental detection limit (log_10_ CFU/lung = 0.7–0.9). Infections were performed in all cases in groups of at least five mice per strain and/or time point (*n* ≥ 5).

We first studied the survival of the *M. pneumoniae* wild-type (WT) strain in lungs. CD1 mice were inoculated intratracheally (i.t.) or intranasally and the bacterial load was determined in lung and bronchoalveolar lavage fluid (BALF) at 2 d postinfection (d.p.i.) (Extended Data Fig. 1a). As the highest bacterial load was detected in lung samples via the intratracheal route, we used intratracheal administration for the remaining experiments. In lungs, the bacterial load decreases at 4 d.p.i. and 14 d.p.i. compared with 2 dpi (2 and 4(log_10_), respectively) (Extended Data Fig. 1b,c), indicating that *M. pneumoniae* is cleared at 14 d.p.i.

Next, we sought to engineer an improved *M. pneumoniae* strain by removing pathogenic genes. The following genes have been suggested as being responsible for *M. pneumoniae* pathogenesis: *mpn372* which encodes the community-acquired respiratory distress syndrome toxin^48,59^; *mpn133* which encodes a lipoprotein with cytotoxic nuclease activity^49^; *mpn453* which encodes the P30 adhesin protein^60^; and *mpn051* which encodes the glycerol-3-phosphate dehydrogenase/oxidase (GlpD or GlpO)^47^. We previously showed that the Δ*mpn051* strain grows poorly in vitro^46^, because the GlpD enzyme is needed for *M. pneumoniae* to use phosphatidylcholine as a carbon source in the lungs^61^. Also, recently we showed that an *M. pneumoniae* strain harboring deletions in the *mpn372* and *mpn133* genes (CV2 strain) was attenuated in a mammary gland infection model^62^. As attenuation can differ in the respiratory tract, and other factors such as adhesion could affect virulence, we characterized the deletion of *mpn453* as an additional gene to be removed to obtain an attenuated lung chassis. In the present study, we observed that the nonadherent Δ*mpn453* strain has a significant reduction of colony-forming units (c.f.u.) recovered at 2 or 4 d.p.i. in mouse lungs compared with the WT strain (Extended Data Fig. 1b), suggesting that attachment to the epithelium is critical for maintenance of *M. pneumoniae* in the lung. Thus, as a compromise between attenuation and maintenance in lungs, we selected, as an attenuated chassis for lung therapy, the CV2 strain, which harbors deleted *mpn372* and *mpn133* genes and WT *mpn453* and *mpn051* genes.

We tested the potential of lung colonization of the CV2 chassis compared with the WT strain by infecting animals i.t. with 1 × 10^7^ c.f.u.(Extended Data Fig. 1c and Table 1). We obtained similar bacterial counts for WT and CV2 strains at 2, 4 and 14 d.p.i., indicating that deletion of *mpn372* and *mpn133* genes did not affect the CV2 capability of colonizing the lung.

**Table 1:** Dose–response of WT or CV2 *M. pneumoniae* strain lung infection

	WT				CV2			
Inoculation dose (c.f.u. per mouse)	10^4^	10^6^	10^7^	10^8^	10^4^	10^6^	10^7^	10^8^
*n* = mice infected/total	1/7	7/7	5/5	7/7	0/7	5/7	6/6	5/5
log_10_(c.f.u. per lung) (mean ± s.d.)	0.9 ± 0.2	3.3 ± 0.9	3.6 ± 0.9	6.4 ± 0.7	0.8 ± 0.07	2.4 ± 1.1	4.0 ± 0.7	5.9 ± 0.7

CD1 mice (*n* = 5–7) were inoculated i.t. with increasing doses of WT or CV2 *M. pneumoniae*. At 4 d.p.i., the ratio of infected mice and the log_10_(c.f.u. per lung) (log_10_(mean ± s.d.)) were determined.

### Lung lesions and inflammatory response induced by the CV2 strain

**Extended Data Fig. 2:**
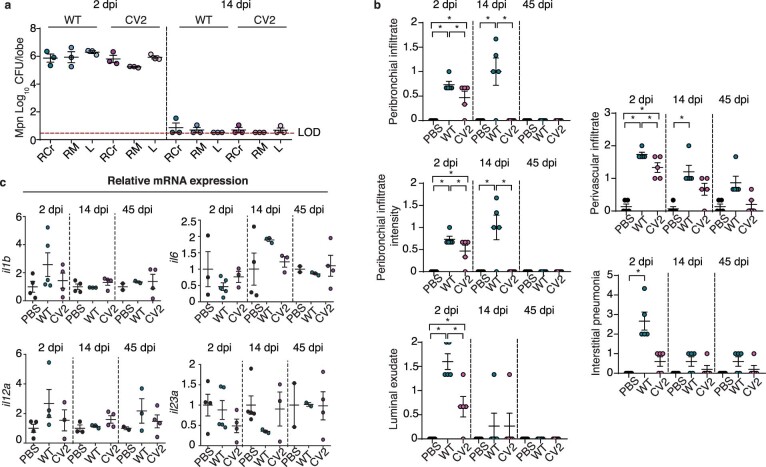
Analysis of the lungs of the animals inoculated with WT or CV2. a, CFUs of the strains WT or CV2, at 2 or 14 days post-inoculation (dpi), found in three major lung lobes (RCr,right cranial; RM, right medium; and L, left. N = 3 animals per group). LOD = limit of detection. Data are expressed as mean ± SD. b, Scoring of the individual parameters analyzed in the histopathologic study of lung sections from mice inoculated with PBS, WT or CV2, analysed at 2, 14 or 45 dpi. See Methods for a detailed description of the histopathological scoring system used. Data are presented as mean values ± SD. Statistical comparisons were performed with One-way ANOVA + Tukey’s multiple comparison test; *P < 0.05. c, Pulmonary inflammatory response to *M pneumoniae* is independent of *il1b*, *il6*, *il-12a* or *il23a*. Gene expression was analysed by RT-qPCR at 2-, 4- and 45 dpi from lungs inoculated with PBS or with WT or CV2 *M*. *pneumoniae* strains. Data are presented as mean values ± SD. Each animal is represented as an individual plot (At 2 dpi: PBS n = 4; CV2 n = 5; CV2_HA_P1 n = 4; at 14 dpi, PBS n = 5; CV2 n = 4; CV2_HA_P1 n = 4 M; at 45 dpi: PBS n = 2; CV2 n = 4; CV2_HA_P1 n = 4).

To compare the response induced by CV2 and WT strains, we inoculated mice with a dose of 1 × 10^7^ c.f.u. and then analyzed lungs at 2, 14 and 45 d.p.i. We determined the bacterial load in the lung tissue by quantifying the colony-forming units. In addition, lesions and immune response were evaluated by histopathology and cytokine profile, respectively. Of note, we found no statistical differences in the recovered colony-forming units between strains at different days postinfection and in different lung lobes (Extended Data Fig. 2a). This result showed a homogeneous distribution of the *M. pneumoniae* strain when inoculated i.t. and corroborated that deletion of genes in the CV2 strain did not affect its lung survival rate.

**Fig. 1:**
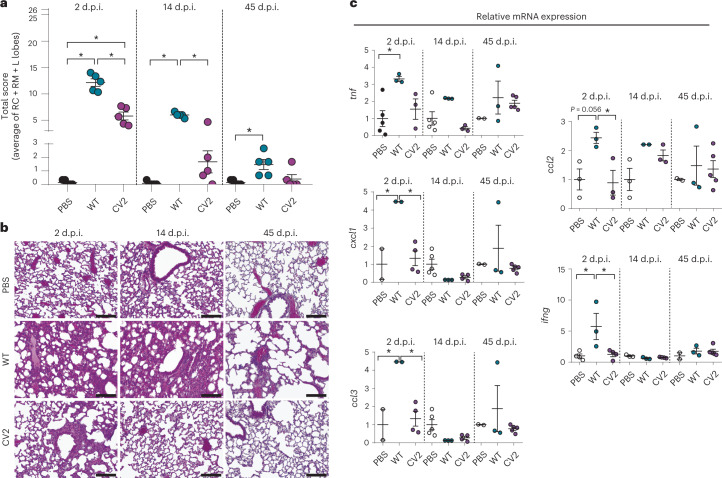
Tissue lesions and inflammatory response of lungs infected with *M. pneumoniae* WT and CV2 strain. CD1 mice (*n* = 5) were inoculated i.t. with *M. pneumoniae* CV2, WT or PBS (as control), and lungs were analyzed at 2, 14 and 45 d.p.i. **a**, Lung lesion evaluation, expressed as the total final score of the histological analysis performed on three major lobes. Each datapoint represents the average of the total score of the right cranial (RC), right middle (RM) and left lobes (L lobes). The mean values of each experimental group ± s.d. are indicated. ^*^P < 0.05 by one-way ANOVA + Tukey’s multiple-comparison test. For a detailed description of the scoring system used in the histopathological analysis, see Methods. **b**, Representative H&E-stained lung sections (200×) from the left lobe. Scale bar, 100 µm. **c**, Gene expression of inflammatory markers, assessed by RT–qPCR. Data are shown as mean ± s.d. of 2^−ΔΔ*Ct*^. *P < 0.05 by one-way ANOVA + Tukey’s multiple-comparison test.

We then evaluated pulmonary lesions by histopathological analyses of three major lung lobes (right cranial, right middle and left lobe) using five parameters: (1) presence of peribronchial/peribronchiolar inflammatory infiltrate (%); (2) intensity of peribronchial/peribronchiolar inflammatory infiltrate; (3) intensity of bronchial/bronchiolar luminal exudate; (4) presence of perivascular inflammatory infiltrate (%); and (5) interstitial pneumonia intensity^63^. Based on these parameters, and as previously described^62,63^, a final total score of up to 26 points was calculated as a global indicator of the lung lesion (Fig. 1a,b, Extended Data Fig. 2b and Supplementary Table 1). More details on the scoring system are given in Methods.

At 2 d.p.i., the total score indicated significantly milder lesions in the CV2 mice compared with the WT infected group (5.8 and 12.1 average points, respectively), with less peribronchiolar and perivascular inflammation and markedly less interstitial inflammation. At 14 d.p.i., the CV2 group showed no significant differences compared with the phosphate-buffered saline (PBS) control group, whereas the WT had remnant tissue injuries related to peribronchiolar, perivascular and interstitial inflammation (score of 6 in the WT versus 1.7 in CV2). At 45 d.p.i., the analyses indicated resolution of lung lesions in both CV2 and WT administered groups (except for perivascular infiltrate, which, although statistically not significant, was still high).

We next studied the induced inflammatory response by evaluating the expression of inflammatory markers in lungs by reverse transcription quantitative PCR (RT–qPCR; Fig. 1c, Extended Data Fig. 2c and Supplementary Table 2). The following panel of genes was analyzed: *il1b*, *il6*, *il12a*, *il23a*, *ifng*, *tnf*, *ccl2*, *ccl3* and *cxcl1*. We did not observe a significant induction of the *il1b*, *il6*, *il12a* and *il23a* genes by WT and CV2 (Extended Data Fig. 2c). In general, the response of the remaining markers was not high, although differences between CV2 and WT were observed. At 2 d.p.i., the WT strain induced expression of the inflammatory markers *tnf*, *cxcl1*, *ccl3*, *ccl2* and *ifng* compared with the PBS control mice, whereas CV2 did not induce any marker compared with WT mice. At 14 and 45 d.p.i., and in agreement with the histopathological analysis, the inflammatory response in both WT and CV2 lungs decreased to levels observed in PBS control mice (Fig. 1c).

Taken together, these results demonstrated that CV2 was attenuated in the lungs compared with the WT *M. pneumoniae* strain, underscoring it as a strong candidate for further engineering as a chassis to treat respiratory diseases.

### Engineering an *M. pneumoniae* strain to dissolve *P. aeruginosa* biofilms

**Fig. 2:**
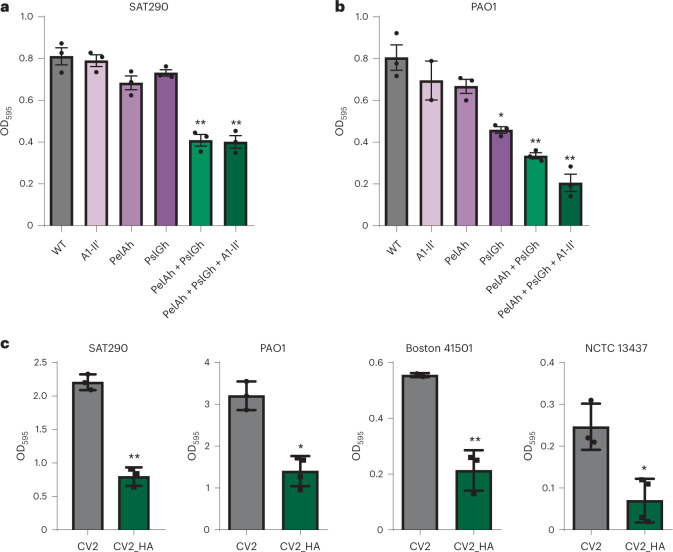
Biofilm dispersion activity of *M. pneumoniae* strains. The activity of the supernatants from *M. pneumoniae* strains against biofilms of the indicated *P. aeruginosa* strains (SAT290, PAO1, Boston 41501, NCTC3437) was assessed by Crystal Volet assay. Briefly, *Pseudomonas* biofilms were generated by seeding in 96-well plates and incubating at 37 °C for 24 h. Biofilms were then treated at 37 °C for 4 h with *M. pneumoniae* supernatants to allow the activity of the dispersal enzymes. More details are given in Methods. **a**,**b**, Biofilm dispersal activity of the supernatants of *M. pneumoniae* strains expressing the indicated heterologous proteins, tested against biofilms of SAT290 (a) or PAO1 (b) strains. **c**, Biofilm dispersal activity of the supernatants of CV2 and CV2_HA. Data are shown as the mean of three independent experiments ± s.d. ^*^P < 0.05, ^**^P < 0.01 by two-sided Student’s *t*-test compared with the control strain. For details (for example, inoculum, growth and time), see Methods.

**Extended Data Fig. 3:**
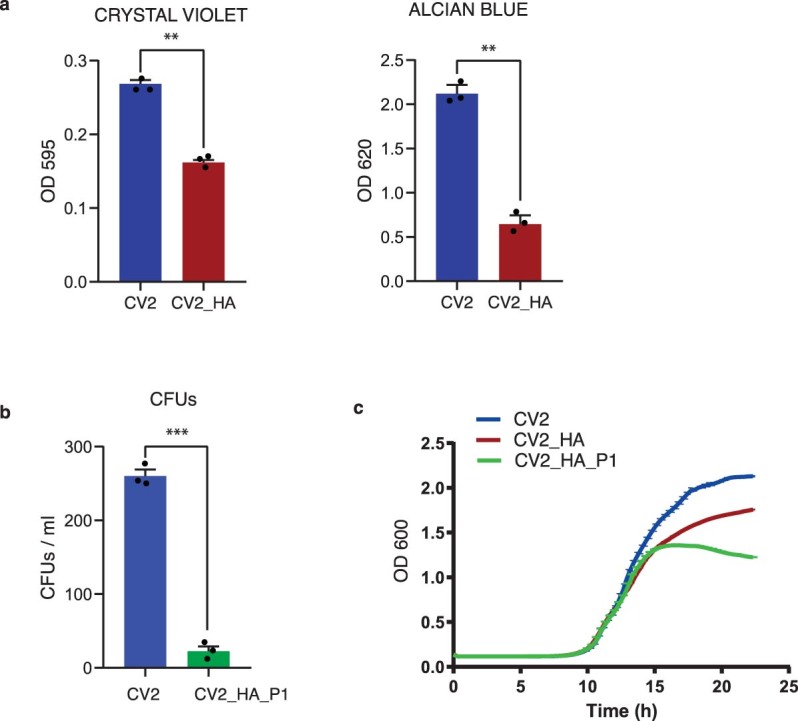
Effect of CV2_HA on *P. aeruginosa* PAO1 biofilm and growth. a, Dispersal activity of CV2_HA on PAO1 biofilm, quantified after staining with crystal violet or Alcian blue. Pseudomonas biofilms were generated by seeding in 96-well plates and incubating at 37 °C for 24 h. Biofilms were then treated at 37 °C for 4 h with *M. pneumoniae* CV2_HA supernatants, to allow the activity of the dispersal enzymes. The remaining biofilm was stained with crystal violet or Alcian blue and quantified with a plate reader at the indicated absorbances (OD 595 or OD 620). Three independent experiments performed. Data are presented as mean values ± SD. **P = 0.009 and P = 0.007, two-sided *t*-test. b. Activity of CV2_HA_P1 on PAO1 biofilm, quantified as CFUs/ml. Pseudomonas biofilms were generated by seeding in 96-well plates and incubating at 37 °C for 24 h. Biofilms were then treated at 37 °C for 4 h with the indicated supernatants, and then the remaining biofilm was resuspended in PBS and seeded on Pseudomonas agar plates. Three independent experiments performed. Data are presented as mean values ± SD. ***P < 0.001, two-sided *t*-test. c, Growth curves of *P. aeruginosa* strain PAO1 in presence of supernatant from CV2, CV2_HA or CV2_HA_P1. Results from triplicate samples are shown. Data are presented as mean values ± SD. Details of the assay are given in the Methods.

To introduce properties into our chassis to treat pulmonary infectious diseases, we designed and characterized an optimal genetic system for dispersing *P. aeruginosa* biofilms, which is one of the main pathogenic bacteria causing VAP. As the *P. aeruginosa* biofilm is mainly composed of the polysaccharides Pel, PsI and alginate, we engineered the WT strain with a genetic cassette expressing three different enzymes that target these three polysaccharides, namely: the glycoside hydrolases PelAh^64^ and PslGh^55^, and the A1-II′ alginate lyase^56^, fused to a peptide for secretion (MPN142_OPT)^62^ (European patent 16706622.4; Supplementary Table 3). First, we confirmed the expression of PelAh, PslGh and A1-II′ in the cell lysate and supernatant of CV2_HA by mass spectroscopy (MS; Supplementary Table 4). We then tested the antibiofilm activity of the supernatants of the different *M. pneumoniae* strains expressing either single enzymes or some of the possible combinations of enzymes against *P. aeruginosa* by Crystal Violet assay. We found that the *M. pneumoniae* strain engineered with a combination of the three enzymes showed the best dispersal activity against biofilms formed by *P. aeruginosa* strains SAT290 and PAO1 (Fig. 2a,b). We obtained similar results when using Alcian Blue as an alternative biofilm-staining method (Extended Data Fig. 3a). Hence, we included PelAh, PslGh and A1-II′ in the attenuated CV2 strain, CV2_HA. Finally, we confirmed the antibiofilm activity of CV2_HA in a panel of clinical *P. aeruginosa* strains (Fig. 2c). These results demonstrated that the *M. pneumoniae* strain CV2, which expresses and secretes the glycoside hydrolases PelAh and PslGh and the A1-II′ alginate lyase, degrades *P. aeruginosa* biofilms in vitro.

### Addition of antimicrobial activity to CV2_HA

**Fig. 3:**
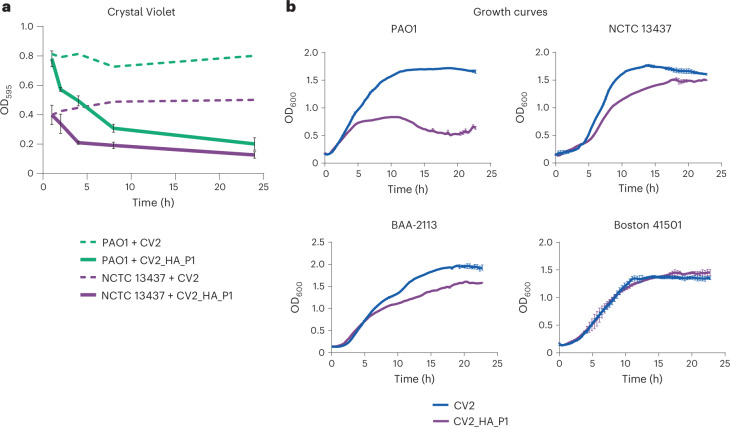
Biofilm dispersion and antimicrobial activities of the CV2_HA_P1 strain in vitro. **a**, Biofilm dispersal activity of the supernatant of strains CV2 (dashed lines) or CV2_HA_P1 (solid lines), assessed by Crystal Violet assay. *Pseudomonas* biofilms were generated in 96-well plates at 37 °C for 24 h and then treated at 37 °C with the indicated supernatants. Data are shown as the mean of three independent experiments ± s.d. with three technical replicates at each timepoint. **b**, Growth curves of different *P. aeruginosa* strains treated with supernatants from CV2 or CV2_HA_P1. Absorbance of the culture (OD_600_) was measured every 20 min with a TECAN reader over 24 h. Errors bars indicate the s.d. of four replicates. For details (for example, inoculum, growth and time), see Methods.

**Extended Data Fig. 4:**
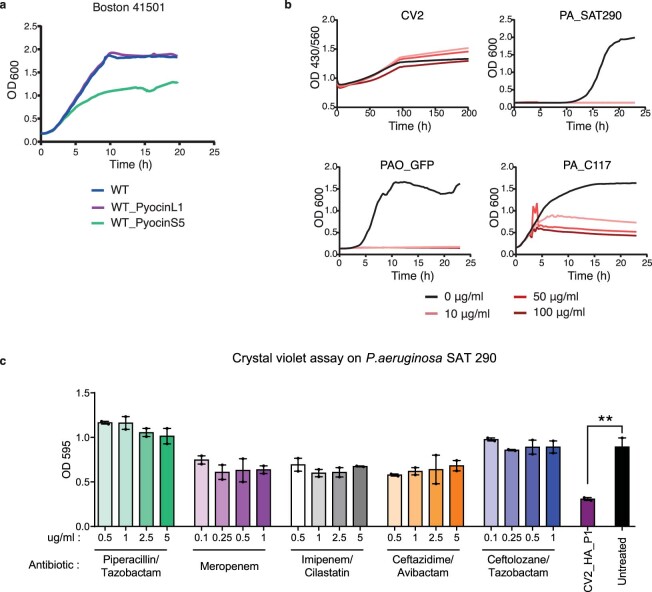
Study of effect of antibiotics on growth curves and biofilms formation. a, Effect of treatment with the WT *M. pneumoniae* strain expressing pyocin L1 or pyocin S5 on the growth of the *P. aeruginosa* Boston 41501 strain. b, Growth curves of CV2 and different *P. aeruginosa* strains (SAT290, PAO1 or C117) in the presence of different doses of the antibiotic combination piperacillin/tazobactam. Growth was measured by absorbance determination at OD 430/560 or OD 600. c, Crystal violet assay measuring *P. aeruginosa* SAT290 biofilm degradation using different antibiotics at different concentrations. Two independent experiments performed. Data are presented as mean values ± SD. **P = 0.004, two-sided *t*-test. No significant effects on biofilm degradation were observed as compared to the untreated control. Details of the assays are given in the Methods.

The ideal engineered *M. pneumoniae* strain to treat VAP should combine biofilm dispersal and antimicrobial activity. To introduce antimicrobial activity, we engineered CV2_HA to express the bacteriocin pyocin L1 (CV2_HA_P1), which was previously shown to kill some *P. aeruginosa* strains, including PAO1 (ref. ^57^). First, we confirmed the capacity of the cell-free supernatant of CV2_HA_P1 to dissolve biofilms formed by two different *P. aeruginosa* strains by Crystal Violet (Fig. 3a) and also the colony-forming unit count (Extended Data Fig. 3b). Then, we characterized the antimicrobial properties of CV2_HA_P1 on four different *P. aeruginosa* strains. We found that CV2_HA_P1 inhibited the growth of PAO1 and showed moderate activity against NCTC13437 and BAA-2113 strains but not against the Boston 41501 strain (Fig. 3b and Extended Data Fig. 3c). To diversify the antimicrobial spectrum, we expressed pyocin S5 (ref. ^55^) instead of pyocin L1, which led to growth inhibition of the *P. aeruginosa* Boston strain (Extended Data Fig. 4a).

These results demonstrated that (1) the strain CV2_HA_P1 displayed both antibiofilm and antimicrobial activities against *P. aeruginosa* and (2) the antimicrobial activity of the engineered *M. pneumoniae* can be modulated to act against specific *P. aeruginosa* strains by introducing different antimicrobial pyocins.

### Effects of CV2_HA_P1 in ETTs from VAP patients

As *M. pneumoniae* lacks a cell wall, we speculated that it could be used in combination with antibiotics targeting the peptidoglycans of the cell wall of both Gram-positive and Gram-negative bacteria. To test this hypothesis, we evaluated the effect on the growth of *M. pneumoniae* and different *P. aeruginosa* strains of antibiotics commonly used in clinics (Table 2 and Extended Data Fig. 4b). As expected, we found that no antibiotics that target the cell wall killed the CV2_HA strain, whereas all the antibiotics were active against most *P. aeruginosa* strains (Table 2). Of note, although the antibiotics did not dissolve *P. aeruginosa* biofilms to any significant degree, incubation with the CV2_HA_P1 strain effectively dissolved the biofilm (Extended Data Fig. 4c).

**Table 2:** Susceptibility of *M. pneumoniae* and *P. aeruginosa* strains SAT290, PAO1 and C117 to different antibiotics

		*M. pneumoniae* CV2_HA		*P. aeruginosa* SAT290		PAO1		*P. aeruginosa* C117	
**Antibiotic**	**Target**	**Nonlethal**	**Lethal**	**Nonlethal**	**Lethal**	**Nonlethal**	**Lethal**	**Nonlethal**	**Lethal**
Piperacillin/tazobactam	Cell wall	500	Nonlethal	ND	5	50	100	200	500
Ciprofloxacin	DNA gyrase	ND	50	ND	50	ND	50	50	100
Levofloxacin	DNA gyrase	ND	20	ND	20	20	200	20	200
Meropenem	Cell wall	500	Nonlethal	ND	1	1	10	10	100
Imipenem/cilastatin	Cell wall	500	Nonlethal	ND	5	5	30	5	300
Amikacin	Ribosome	ND	10	10	100	ND	10	10	100
Ceftacidime/avibactam	Cell wall	15	Nonlethal	0.5	15	5	15	15	ND
Ceftolozane/tazobactam	Cell wall	15	Nonlethal	ND	0.5	0.5	5	15	ND

Inoculum, growth and time are detailed in Methods. Numbers in the table represent the concentrations tested (µg ml^−1^) of each antibiotic. ND, Not determined.

To evaluate the efficacy of our CV2_HA_P1 strain in dissolving in vivo-formed biofilms, we treated sections of ETTs obtained from VAP patients receiving mechanical ventilation in the ICU (see Methods). After a median of 11 d of MV, *P. aeruginosa* biofilms were observed on the ETTs, with bacterial loads of approximately 2.57 (2.20–4.81) log_10_(c.f.u. ml^−1^). The *P. aeruginosa* strains in the ETTs showed resistance to meropenem (100%), imipenem (100%), aztreonam (100%), amikacin (66%) and ciprofloxacin (33%), but were susceptible to colistin, piperacillin/tazobactam, tobramycin and ceftazidime. Multilocus sequence-type analysis of *P. aeruginosa* identified the ST109 and ST259 strains, which are allocated to the clonal complexes 253 and 2044. Complex 253 was previously identified from patients on the ICU^65^ and the complex 2044 was found in patients with bronchiectasis.

**Fig. 4:**
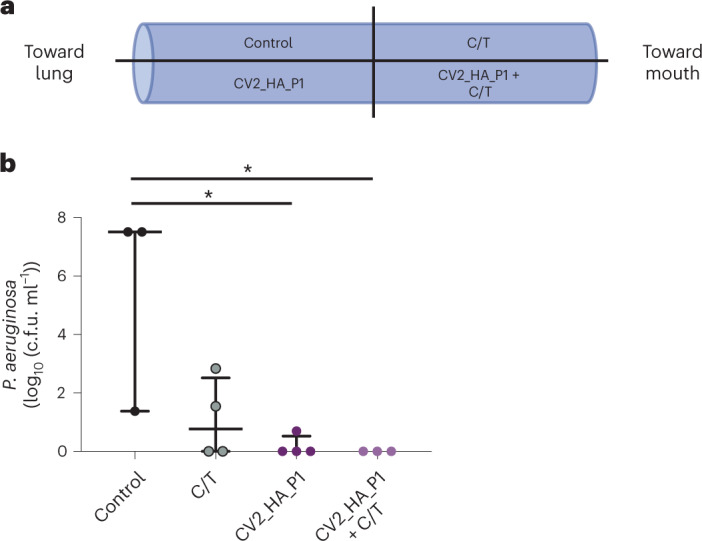
Dispersion of biofilms of ETTs from patients with VAP. **a**, Schematic representation of ETT slices from patients in the ICU who received MV. The 4-cm ETTs were taken from the distal part (for example, the first 10 cm closest to the patient’s lungs) and were sliced into four hemisections. One hemisection was used for each treatment arm: control (Hayflick medium alone without treatment), CV2_HA_P1 (~1 × 10^8^ cells), C/T (5 µg ml^−1^) or CV2_HA_P1 + C/T. **b**, Effect on the *P. aeruginosa* load on ETT biofilm from patients who received MV (see Methods). Significant differences between groups were found: *P* = 0.049 by two-sided Kruskal–Wallis test. The median and the IQR load (log_10_(c.f.u. ml^−1^)) of each treatment group were: (1) control (*n* = 3): 7.51 (4.44–7.51); (2) C/T (*n* = 4): 0.77 (0.00–2.52); (3) CV2_HA_P1 (*n* = 4): 0.00 (0.00–0.52); and (4) CV2_HA_P1 + C/T (*n* = 3): 0.00 (0.00–0.00). The *P* values for pairwise comparisons between groups (Wilcoxon’s signed-rank test) are indicated when significant (^*^P < 0.05). The level of significance for pairwise comparisons was *P* = 0.008.

We included 14 of the 16 ETT sections in the final analysis, distributed as follows in each treatment group: control (*n* = 3), ceftolozane/tazobactam (C/T) (*n* = 4), CV2_HA_P1 (*n* = 4) and CV2_HA_P1 + C/T (*n* = 3; Fig. 4a); the remaining two samples were discarded because no *P. aeruginosa* counts could be obtained due to overgrowth of *Proteus* spp. After a 24-h incubation, the *P. aeruginosa* load showed significant differences between the control and the treated groups (Fig. 4b): ETT biofilms treated with antibiotics reduced the *P. aeruginosa* load and this reduction was even more substantial with the CV2_HA_P1 alone or in combination with the C/T antibiotics. These results demonstrate that CV2_HA_P1 has broad-spectrum activity against biofilms formed by different multidrug-resistant *P. aeruginosa* clinical strains.

### Efficacy of CV2_HA_P1 in a murine lung infection model

**Extended Data Fig. 5:**
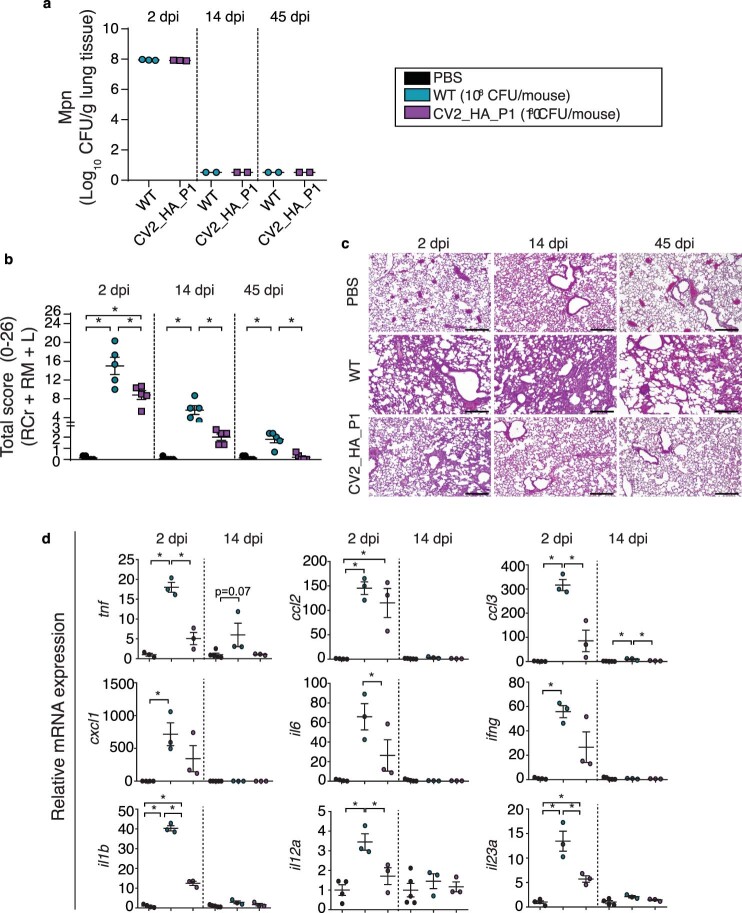
Tissue lesions and inflammatory response of lungs infected with *M. pneumoniae* WT and CV2_HA_P1 strain at 10^8^ CFUs. CD1 mice were inoculated intratracheally with *M. pneumoniae* CV2_HA_P1, WT or PBS (as control), and lungs were analysed at 2-, 14- and 45 dpi. a, CFUs of the strains WT or CV2_HA_P1, at 2, 14 or 45 days post-inoculation (dpi) recovered in lungs (n = 3, 2 and 2, respectively). b, Lung lesions evaluation, expressed as the total final score of the histological analysis performed on three major lobes, with five animals per group (each data point is the average of the total score of the Right Cranial, Right Middle and Left of each animal). Data are presented as mean values of each group ± SD. *P < 0.05; One-way ANOVA + Tukey’s multiple comparison test. For detailed description of the scoring system used in the histopathological analysis, see Methods. c, Representative H&E-stained lung sections (100⋅, scale bar 100 µm) from the left lobe, obtained using a digital camera (MC170 HD, Leica) connected to an optical microscope (DM2000, Leica) using a commercial software (Leica Application Suite, version 4.6.0). d, Gene expression of inflammatory markers, assessed by RT-qPCR. Data are shown as mean ± SD of 2^-ΔΔCt^. Each animal is indicated as an individual plot. Each animal is represented as an individual plot (PBS, n = 4; CV2, n = 3; CV2_HA_P1, n = 3). *P < 0.05, One-way ANOVA + Tukey’s multiple comparison test.

To study the efficacy of CV2_HA_P1 in vivo, we first tested its toxicity by inoculating mice with 1 × 10^8^ c.f.u. and analyzing lungs at 2, 14 and 45 d.p.i. by histopathology and cytokine measurement (Extended Data Fig. 5 and Supplementary Tables 5 and 6).

At 2 d.p.i., the same bacterial load was recovered in lungs of WT and CV2_HA_P1 strains; however, the CV2_HA_P1 lungs showed significantly fewer alterations compared with the WT group (*P* < 0.05). At 14 and 45 d.p.i., no colony-forming units were detected in CV2_HA_P1 lungs and the total score of the histopathology was not significantly different from the PBS controls; in stark contrast, WT infected lungs still presented tissue lesions (Extended Data Fig. 5a–c and Supplementary Table 5). Study of inflammation markers corroborated the resolution of lesions and the attenuation of the engineered CV2_HA_P1 strain (Extended Data Fig. 5d and Supplementary Table 6).

**Extended Data Fig. 6:**
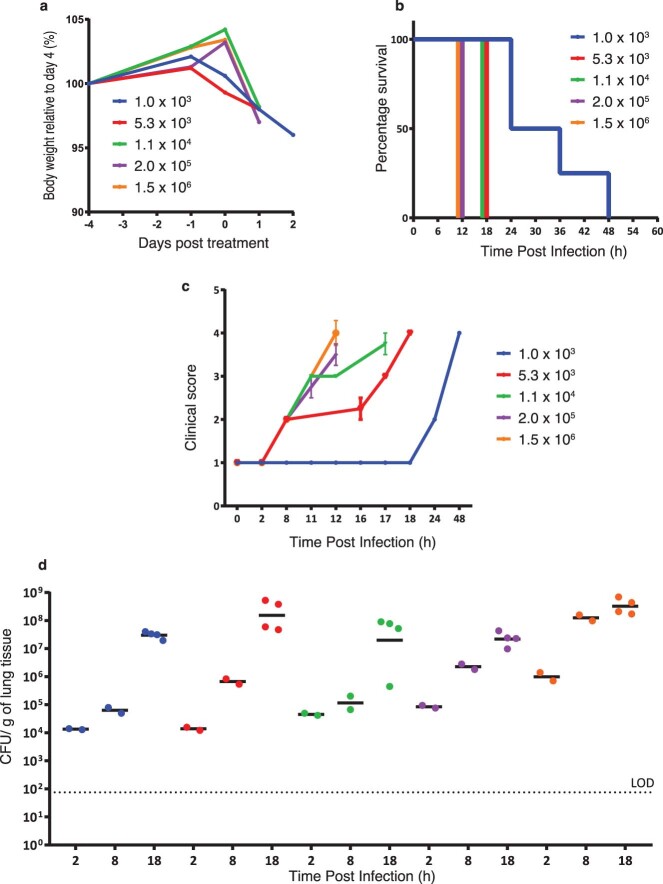
Set up of acute model of *P. aeruginosa* infection. CD1 mice were IT infected with different amounts of P. aeruginosa PAO1 (1.0× 10^3^, 5.3× 10^3^, 1.1× 10^4^, 2.0× 10^5^ or 1.5× 10^6^ CFU/mouse). a, Evaluation of body weight of mice infected with different doses of *P. aeruginosa* PAO1. b, Endpoints at which mice were euthanized by pentobarbitone overdose. c, Clinical score (see Methods) of the mice infected with different doses of *P. aeruginosa* PAO1 (Five animals per groups. Data are presented as mean values ± SD.). d, Number of CFUs recovered from the lung of mice after inoculation with different doses of PAO1 at different time points post-inoculation (2, 8 or 18 hpi. N = two or four animals per group, as indicated by the data points). LOD = Limit of detection.

Next, we established a murine model of acute lung infection using PAO1. We immunosuppressed mice with cyclophosphamide and inoculated i.t. different amounts of the *P. aeruginosa* PAO1 strain (*n* = 8 mice for each group). Mouse survival, body weight and clinical conditions were assessed at different time points (Extended Data Fig. 6a–d). Mice infected with doses >1 × 10^4^ c.f.u. had to be sacrificed or died at 12 h post-inoculation (h.p.i.); mice infected with 5 × 10^3^ and 1 × 10^4^ c.f.u. survive until 18 h.p.i. (Extended Data Fig. 6b), at which point the clinical score was 4 (Extended Data Fig. 6c; see Methods), and mice infected with 1 × 10^3^ c.f.u. survived until 24 h.p.i. with a final clinical score of 2 and died by 48 h.p.i. Based on these results, we decided to use an inoculum of 1 × 10^3^ c.f.u. per mouse of PAO1, which ensured lung colonization of the infected mice without treatment and survival for a longer period, thereby allowing the therapeutic effect of our chassis to be monitored.

**Fig. 5:**
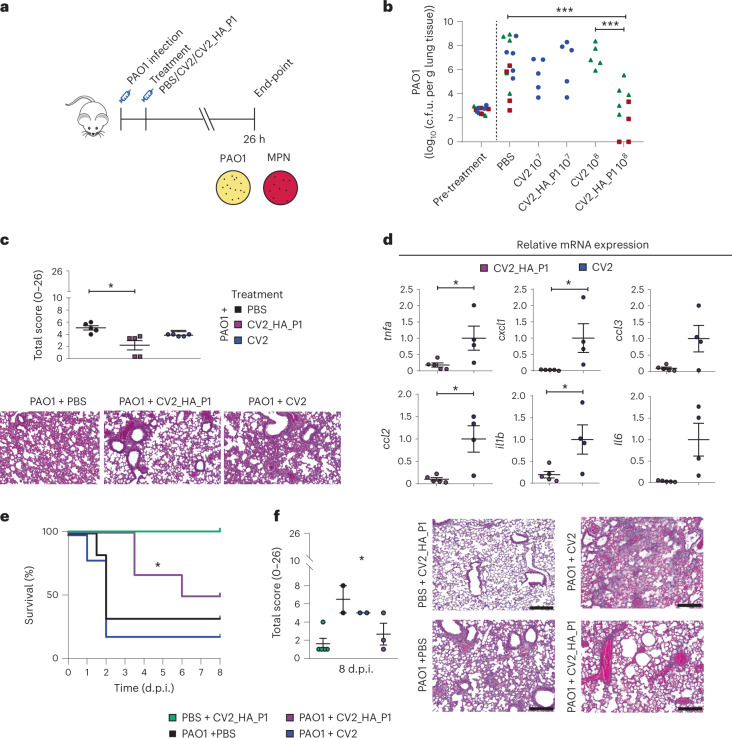
In vivo treatment of mice with acute respiratory PAO1 infection. **a**, CD1 mice immunocompromised with cyclophosphamide and infected i.t. with 1 × 10^3^ c.f.u. of *P. aeruginosa* PAO1. At 2 h.p.i., mice were treated i.t. with 1 × 10^7^ (experiment (exp.) 1) or 1 × 10^8^ (exp. 2 and 3) c.f.u. of CV2_HA_P1 *M. pneumoniae* strains or CV2 strain, or PBS. MPN: *M. pneumoniae*. At 26 h.p.i., mice were sacrificed to determine the PAO1 load in the lungs after the treatments (more details in Methods). **b**, Colony-forming units for *P. aeruginosa* PAO1 found in different treatment conditions. Three independent experiments were performed, indicated by different symbols in the graph: exp. 1 (blue circles), exp. 2 (red squares) and exp. 3 (green triangles). In each experiment, five mice were used per treatment group. Five additional mice were kept untreated and sacrificed to determine the PAO1 load in the lungs before the treatments (pretreatment control). ^***^P < 0.001 by two-sided Student’s *t*-test. **c**, Lung lesion evaluation (total score) of mice infected with PAO1 and treated with the indicated *M. pneumoniae* strains. ^*^P < 0.05 by one-way ANOVA + Tukey’s multiple-comparison test. Histological analysis was performed on three major lobes (right cranial, right middle and left). See Methods for further detailed description of the scoring system used in the histopathological analysis. Below, representative H&E-stained lung sections (100×) from the left lobe. Scale bar, 100 µm. **d**, Relative gene expression of inflammatory markers, assessed by RT–qPCR of lung homogenates. Each animal used is marked as an individual dot (CV2, *n* = 4; CV2_HA_P1, *n* = 5). Data are shown as mean ± s.d. of 2^−ΔΔ*Ct*^. ^*^P < 0.05 by two-sided Student’s *t*-test. **e**, Survival of mice infected with PAO1 and treated with CV2_HA_P1 or controls. ^*^P = 0.0357 by Gehan–Breslow–Wilcoxon test with comparison to CV2 control. **f**, Lung lesions assessed by histological analysis of the lungs of the animals that survived until 8 d.p.i. (*n* = 5 for PBS + CV2_HA_P1 control; *n* = 2 for PAO1 + PBS and for PAO1 + CV2 groups; *n* = 3 for PAO1 + CV2_HA_P1 group). See Methods for details of the scoring. ^*^P = 0.0189 by one-way ANOVA + Bonferroni’s test. Right, representative H&E-stained lung sections (100×) from the left lobe, obtained using a digital camera (MC170 HD, Leica) connected to an optical microscope (DM2000, Leica) using a commercial software (Leica Application Suite, v.4.6.0). Scale bar, 100 µm.

**Extended Data Fig. 7:**
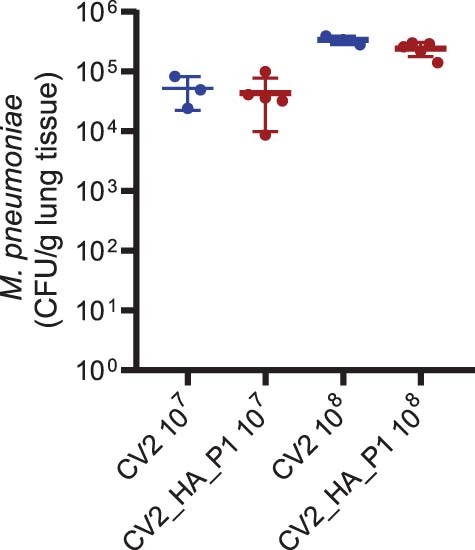
*M. pneumoniae* CFUs detected in lungs of animals of the efficacy assays. Number of CFUs of *M. pneumoniae* strains CV2 and CV2_HA_P1 strains (inoculated at 1× 10^7^ or 1× 10^8^ CFU/mouse) recovered from the lung at 24 hpi. Animals (n = 3 for CV2 controls, and n = 5 for CV2_HA_P1 group) were sacrificed and the lungs were homogenized and serial diluted on Hayflick-agar plates. Data are presented as mean values ± SD.

Next, we studied the efficacy of CV2_HA_P1 in reducing the PAO1 load in the established lung infection model (Fig. 5a). We infected mice with the PAO1 strain, 1 × 10^3^ c.f.u. per mouse (as described above), and treated them at 2 h.p.i. with different amounts (1 × 10^7^ or 1 × 10^8^ c.f.u.) of CV2_HA_P1, CV2 or PBS. All mice in the groups survived to 26 h.p.i., at which point they showed decreased body weight and clinical score 2, but no other signs of clinical deterioration. After sacrifice at 26 h.p.i., we counted the bacterial burden in the lungs. CV2 and CV2_HA_P1 colony-forming units were detected in the lungs (Extended Data Fig. 7), indicating that *M. pneumoniae* can colonize the lungs in the presence of a more severe pathogenic bacterium such as *P. aeruginosa*. The mean burden of PAO1 in the PBS control was 1 × 10^6^ c.f.u. per g of lung tissue. Mice treated with CV2_HA_P1 at 1 × 10^8^ c.f.u. had a significant reduction in the PAO1 load in the lungs compared with the PBS or CV2 control (reduction of 3.65log_10_ and 4.39log_10_, respectively; Fig. 5b). CV2 did not significantly reduce the PAO1 burden in the lungs compared with vehicle or pretreatment control groups at any time. We also studied the histopathology of the lungs at this timepoint. In agreement with the reduction of the PAO1 load, we observed that the total score of lung lesions was significantly lower in the lungs of mice treated with CV2_HA_P1 compared with the controls (Fig. 5c), with less perivascular inflammation and markedly less parenchymal pneumonia. Inflammation markers were also reduced in the lungs of CV2_HA_P1 mice compared with the CV2 group (Fig. 5d). These data indicated that CV2_HA_P1 treatment reduced the PAO1 lung infection in an in vivo model of acute pneumonia.

To study the effect of the CV2_HA_P1 therapy beyond 26 h.p.i., we next analyzed the survival of the mice over days after infection with PAO1 (Fig. 5e). About 50% of mice infected with PAO1 and treated with CV2_HA_P1 survived up to 8 d.p.i. (with a median of 7 d.p.i.), whereas mice treated with the CV2 or PBS control had a median survival of 2 d.p.i. Moreover, histology of mice with PAO1 infection that survived to the 8-d.p.i. timepoint revealed substantially fewer lung alterations in the CV2_HA_P1 group than in the CV2 or PBS control groups (Fig. 5f). These data indicated that CV2_HA_P1 treatment reduced PAO1 lung infection and increased mouse survival.

**Extended Data Fig. 8:**
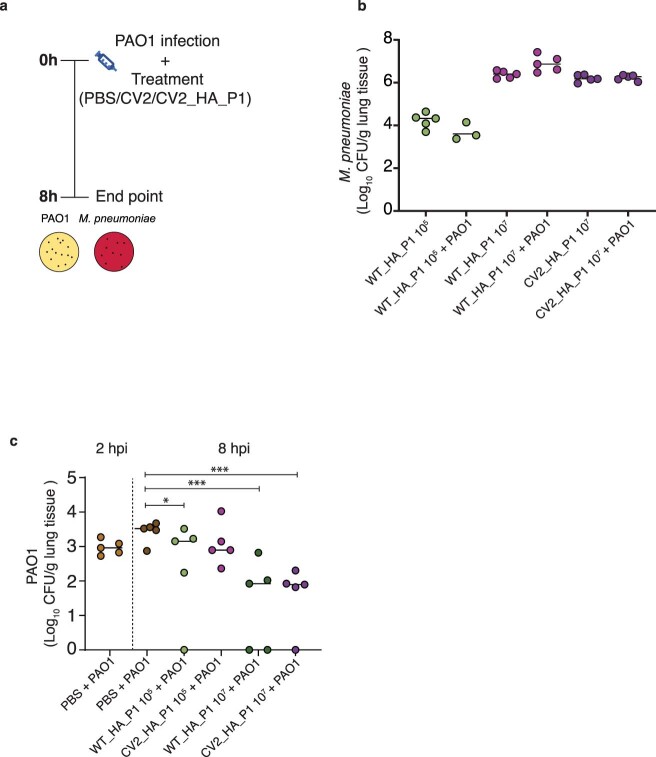
Preventive effect of CV2_HA_P1 on pneumonia induced by PAO1. a, Schematic representation of the experimental design. Two amounts (1× 10^5^ or 1× 10^7^ CFU) of different *M. pneumoniae* strains, alone or in combination with *P. aeruginosa* PAO1 cells, were inoculated intratracheally in the animals (five animals per group). After 8 h, mice were sacrificed, and lungs were homogenized and seeded on Hayflick agar plates to quantify the CFU of *M. pneumoniae* strains (in b) or seeded on Pseudomonas agar to quantify PAO1 (in c). *P < 0.05, ***P < 0.001; *P* values of Kruskal–Wallis *H* test compared to the control at 8 h are indicated.

We also studied the efficacy of CV2_HA_P1 as a prophylactic treatment against PAO1 infection (Extended Data Fig. 8a). We inoculated mice with a mix of *M. pneumoniae* (WT_HA_P1 or CV2_HA_P1; doses 1 × 10^5^ or 1 × 10^7^ c.f.u., respectively) and PAO1, and we studied the progression of PAO1 infection up to 8 d.p.i. *M. pneumoniae* colony-forming units in the lungs were recovered from all groups (Extended Data Fig. 8b). PAO1 colony-forming units were significantly reduced in the lungs of mice treated with 1 × 10^7^ c.f.u. of WT_HA_P1 or CV2_HA_P1 strains compared with nontreated control mice (Extended Data Fig. 8c).

Taken together, these results demonstrated that our engineered *M. pneumoniae* strain CV2_HA_P1 was efficient in treating acute PAO1 infections in a mouse model.

## Discussion

Lung infections, one of the major causes of human mortality, represent an untapped target for bacterial therapeutics. In the present study, we have shown that an engineered strain of the genome-reduced human lung bacterium *M. pneumoniae* (CV2) is attenuated in the lung and can be used to treat respiratory diseases associated with biofilm formation, such as VAP. The CV2 strain produces mild lesions in the acute phase of the lung infection that are resolved, avoiding chronic damage to the lung tissue, having an attenuated inflammation response and being removed spontaneously from the mouse lung after 4 d.p.i.

To demonstrate the potential use of our chassis to treat infectious lung diseases, we introduced genes to dissolve biofilms made by *P. aeruginosa* and to kill this bacterium. The biofilm matrix of *P. aeruginosa* PAO1 comprises DNA, proteins and the polysaccharides Pel and Psl^54–56^; alginate is also a main component of the biofilm of mucoid *P. aeruginosa* strains^12,56^. PelAh and PslGh have been reported as optimal enzymes for degradation of Pel and Psl exopolysaccharides, respectively^55,64^. The alginate lyase enzyme AI-II′, with both poly(M) and poly(G) activities, is effective against biofilms of *P. aeruginosa* mucoid strains^56^. We found that the strain expressing the three enzymes (CV2_HA) was effective in dissolving biofilms formed by different mucoid strains of *P. aeruginosa*. As it was previously reported that combining dispersal and antimicrobial activities could enhance biofilm degradation^56,62^, we engineered *M. pneumoniae* to express active pyocin L1 and pyocin S5. Both pyocins were active and specific for the target strains. We decided to use the *M. pneumoniae* strain expressing pyocin L1 (CV2_HA_P1), which proved to have an effective antimicrobial activity against *P. aeruginosa* PAO1 infection (which we used in the in vivo mouse infection model). We demonstrated the efficacy of our CV2_HA_P1 strain in dissolving biofilms on ETTs from patients in ICU who had received MV for long periods. We also observed that we could rescue the efficacy of standard-of-care antibiotics that were not efficient because of biofilm when combined with CV2_HA_P1, which opens the way for synergistic combinations.

In a mouse model of acute *P. aeruginosa* PAO1 infection, CV2_HA_P1 treatment eliminated the infection at 26 h.p.i. and increased the survival rate of the treated mice. No adverse clinical symptoms were observed related to body weight loss, fever, piloerection or respiratory distress, suggesting that treatment with CV2_HA_P1 at a high single dose was effective without toxicity for up to 8 d.p.i. In addition, CV2_HA_P1 showed a prophylactic effect against *P. aeruginosa* biofilm in vivo, expanding its potential clinical applications.

In conclusion, we have provided evidence that a lung biotherapeutic bacterial strain is effective against biofilm-associated infections in the respiratory tract. We envision that our CV2 chassis could be adapted for the treatment of other infectious and noninfectious lung diseases that require continuous local delivery of therapeutic molecules.

## Methods

### Medium and strain growth conditions

Liquid Hayflick complete medium was prepared by mixing 800 ml of noncomplete medium A (20 g of PPLO broth (Difco, catalog no. 255420), 30 g of Hepes (100 mM final), 25 ml of 0.5% phenol red solution (Sigma-Aldrich, catalog no. P3532)), 200 ml of heat-inactivated horse serum (Life Technologies, catalog no. 26050088), 20 ml of sterile filtered 50% glucose and 1 ml of a 100-mg ml^–1^ stock of ampicillin (final concentration 100 µg ml^–1^, ampicillin sodium salt; Sigma-Alrich, catalog no. A9518). Solid Hayflick 1% agar plates were prepared by mixing 800 ml of noncomplete medium A with 10 g of Bacto Agar (BD, catalog no. 214010) and adding 200 ml of heat-inactivated horse serum, 20 ml of sterile filtered 50% glucose and 1 ml of a 100-mg ml^–1^ stock of ampicillin (final concentration 100 µg ml^–1^).

For *P. aeruginosa* growth curves, strains were grown overnight in Tryptic Soy Broth (TSB) medium at 37 °C with shaking. The next day, the cultures were diluted in TSB to absorbance at 600 nm (OD_600_) = 0.1, and 200 µl was added to 96-well plates and grown at 37 °C with shaking in a TECAN plate reader. Growth was measured as an increase in OD_600_, with values taken every 20 min up to 48 h. For *M. pneumoniae* growth curves, strains were inoculated in Hayflick medium in a T75-cm^2^ flask and grown at 37 °C. After 3 d, cells were scraped from the flasks and resuspended in 1 ml of Hayflick medium. The cell suspension was then diluted 1:200 and 200 µl of this suspension was added to 96-well plates and incubated on static conditions at 37 °C in a TECAN plate reader. Growth was measured as an increase of the ratio between absorbance at 430 nm and 560 nm, with values at 430 nm and 560 nm taken every 2 h for 4 d.

### Plasmids

All plasmids generated in this work were assembled following the Gibson method^63^. When required, IDT Incorporation performed gene synthesis. Oligonucleotides were synthesized by Sigma-Aldrich. Gene amplifications were carried out with Phusion DNA polymerase (Thermo Fisher Scientific). A description of the plasmids is available in Supplementary Table 3. The final sequence of all the plasmids was checked by Sanger sequencing (Eurofins Genomics).

### Generation of *M. pneumoniae* mutant strains

The mutant strain Δ*mpn453* and CV2 (double mutant carrying deletions in both *mpn133* and *mpn372*) genes were constructed in previous work^62^. *M. pneumoniae* strains with genetic platforms were generated by transforming the CV2 and WT strains with vectors described in Supplementary Table 3 by electroporation. After pulsing, cells were selected in T75-cm^2^ flasks containing 25 ml of plain Hayflick medium with selective antibiotics (tetracycline, 2 μg ml^–1^, gentamicin, 100 μg ml^–1^, or chloramphenicol, 20 μg ml^–1^).

### Protein quantification by MS

For the proteome samples of different mutant strains, *M. pneumoniae* strains were grown to the exponential phase of growth. After a medium sample of 2 ml was removed, cells were washed 3× with PBS and collected in 1 ml of PBS by scraping. Cell samples were centrifuged at 14,000*g* for 15 min and the pellet was resuspended in 50 µl of 6 M urea (in 200 mM ammonium bicarbonate). The medium samples were centrifuged at 14,000*g* and then passed over a 0.1-μm filter. The 800-μl sample was concentrated to 50 μl using 3K MWCO columns, and 75 μl of urea in 200 mM ammonium bicarbonate was added to a final concentration of 6 M urea. After 15 min of sonication, all samples were quantified using bicinchoninic acid and then processed for MS. Samples were analyzed by tandem MS combined with liquid chromatography. Briefly, in solution digestion samples were reduced with dithiothreitol (30 nmol, 1 h, 37 °C) and alkylated in the dark with iodoacetamide (60 nmol, 30 min, 25 °C). The resulting protein extract was first diluted 1:3 with 200 mM NH_4_HCO_3_ and digested with 1 µg of LysC (Wako, catalog no. 129–02541) overnight at 37 °C and then diluted 1:2 and digested with 1 µg of trypsin (Promega, catalog no. V5113) for 8 h at 37 °C. The tryptic peptides were then first acidified and desalted with a MicroSpin C18 column (The Nest Group, Inc.). Samples were analyzed using an LTQ-Orbitrap Velos Pro mass spectrometer coupled to an EASY-nLC 1000 (Thermo Fisher Scientific). The sample was loaded on to the 2-cm Nano Trap column (inner diameter 100 μm, 5-μm C18 particles; Thermo Fisher Scientific) and separated by reversed-phase chromatography using a 25-cm column (inner diameter 75 μm, 1.9-μm C18 particles; Nikkyo Technos Co., Ltd.). Chromatographic gradients started at 93% buffer A and 7% buffer B with a flow rate of 250 nl min^−1^ for 5 min and gradually increased to 65% buffer A and 35% buffer B in 120 min. After each analysis, the column was washed for 15 min with 10% buffer A and 90% buffer B. Buffer A is 0.1% formic acid in water and buffer B 0.1% formic acid in acetonitrile. The mass spectrometer was operated in positive ionization mode with nanospray voltage set at 2.1 kV and source temperature at 300 °C. Before the analysis, we performed external calibration of the Fourier transform with Ultramark 1621 and internal calibration with the ion signal of the poly(siloxane) background (*m*/*z* 445.1200). For the MS scans, the data-dependent acquisition (DDA) mode was set at a resolution of 60,000, *m*/*z* range 350–2,000 and detected in the Orbitrap (automatic gain control (AGC) = 1 × 10^6^ and dynamic exclusion of 60 s). The top 20 most intense ions were selected for collision-induced dissociation fragmentation with normalized collision energy of 35% in each cycle of DDA analysis. For the injection, we selected AGC to 1 × 10^4^, 2.0*-m*/*z* isolation window, 10 ms of activation time and 100 ms of maximum injection time. Xcalibur software v.2.2 was used for data acquisition. To eliminate sample residues and ensure the stability of the equipment, we analyzed digested bovine serum albumin (New England Biolabs) between samples^66^. Proteome Discoverer (Thermo Fisher Scientific) software and Mascot^67^ and search engine (Matrix Science) were used for the analysis of the acquired spectra. The data were searched against an *M. pneumoniae* database plus a list of common contaminants^68^ (87,070 entries) and all the corresponding decoy entries. A precursor ion mass tolerance of 7 p.p.m. was used for MS1 level for peptide identification, using trypsin as the enzyme and allowing up to three missed cleavages. For MS2 spectra, ion mass tolerance was established at 0.5. We used oxidation of the methionine and amino-terminal protein acetylation as variable modifications, and carbamidomethylation on cysteine as a fixed modification. The false discovery rate in peptide identification was set to a maximum of 5%.

The ‘precursor ion area detector’ node from Proteome Discoverer (v.2.0) was used for peptide quantification. The obtained values were used to calculate the protein top three areas with the unique peptide for protein ungrouped (Supplementary Table 4). The raw proteomics data have been deposited to the PRIDE^69^ repository with the accession no. PXD037233.

### In vitro biofilm degradation assay

*M. pneumoniae* was grown in a T25-cm^2^ flask for 3 d with 5 ml of Hayflick medium without antibiotics and then the conditioned supernatant was filtered with 0.33-µm sterile syringe filters. *P. aeruginosa* strains were grown overnight in Erlenmeyer flasks (20 µl stock in 20 ml of TSB) at 37 °C on shaking at 600 r.p.m., and then diluted to an OD_600_ of 0.15 in TSB. Diluted *Pseudomonas* culture (100 µl) was then added in triplicate to sterile 96-well, polystyrene microtiter plates. Cells were incubated statically at 37 °C for 24 h to allow for biofilm formation. Biofilms were washed with PBS the following day to remove nonadherent cells and TSB medium. Treatments of 50–100 µl of *M. pneumoniae*-conditioned filtered medium were added to the wells (using triplicates or more) and plates were incubated at 37 °C for 4 h. After incubation, wells were washed with PBS, stained with 150 µl of 0.1% (w:v in water) Crystal Violet (Sigma-Aldrich) for 10 min, or with 150 µl of 0.1% (w:v in 3% acetic acid) Alcian Blue (Sigma-Aldrich), and washed 3× with PBS. The dye was solubilized by addition of 100 μl of 95% (v:v) ethanol and incubated for 10 min. Absorbance was measured at 595 nm (for Crystal Violet) or 620 nm (for Alcian Blue) using a TECAN plate reader.

To measure the impact of *M. pneumoniae* supernatants on the number of PAO1 cells, biofilms were formed in 96-well plate and treated as described above. After incubation, supernatants were removed and the attached cells were recovered in 200 µl of PBS, serially diluted, seeded on *Pseudomonas* agar plates and incubated at 37 °C for 24 h. The colony-forming units were counted in two independent experiments with three technical triplicates each.

### In vitro antimicrobial activity test

The antimicrobial activities of the supernatant of *M. pneumoniae* strains expressing pyocins were tested in a growth curve. *M. pneumoniae* was grown in a T25-cm^2^ flask to confluence (3–4 d at 37 °C, 5% CO_2_) with 5 ml of Hayflick medium without antibiotics, and then the supernatant medium was filtered with 0.33-µm sterile syringe filters. *P. aeruginosa* strains were grown overnight in Erlenmeyer flasks (20 µl of stock in 20 ml of TSB) at 37 °C with shaking and then diluted to an OD_600_ of 0.1 in TSB. Diluted *Pseudomonas* culture (180 µl) was mixed with 20 µl of filtered *M. pneumoniae* supernatant into sterile, 96-well, polystyrene microtiter plates. All conditions were tested at least in triplicate. Plates were incubated in a TECAN reader at 37 °C with shaking and the OD_600_ was measured every 20 min.

### Mice experiments

#### Ethics

The animals used in all the studies were CD1 mice (female and male, weight 18–22 g and aged 4–6 weeks), purchased from Charles River Laboratories, and specific pathogen free. Group animal size were computed with G*Power software. The number of animals used in each experiment is specified in the figure legends.

The experiments, aimed to set up the routes of administration, the *M. pneumoniae* doses and the CV2 strain safety, were performed either at the Institute of Agrobiotechnology facilities (registration no. ES/31-2016-000002-CR-SU-US) or at the Barcelona Biomedical Research Parc (PRBB) facilities (registration no. B9900073). All the procedures involving animals were legislated for by the European Directive 86/609/EEC and the National law (Real Decreto 53/2013), in accordance with the FELASA and ARRIVE guidelines and the agreement of the Universidad Pública de Navarra (UPNa), the PRBB Animal Experimentation Committee (Comité de Ética, Experimentación Animal y Bioseguridad) and the local government authorization. The experiments aimed at studying the efficacy in vivo were performed in the UK under the Home Office Licence PA67E0BAA with local ethical committee clearance (Animal Welfare and Ethical Review Body). All the experiments were performed in dedicated Biohazard 2 facilities (this site holds a Certificate of Designation). On receipt at the facility, animals were housed in sterilized, individually ventilated cages connected to HEPA (high-efficiency particulate absorbing)-filtered sterile air and allowed to acclimatize for at least 7 d. Mice always had free access to food and water (sterile) and aspen chip bedding. The room temperature was 22 °C ± 1 °C, with a relative humidity of 60% and maximum background noise of 56 dB. Mice were exposed to 12:12 h light:dark cycles. Mice were monitored at least once daily for their clinical condition and assessed for their clinical score. The clinical score was given as follows: 1 = no deterioration in clinical condition, may include slight piloerection, normal activity; 2 = slight piloerection, slightly hunched, dehydrated, weight loss <20%; 3 = piloerection, moderate intermittent hunching, dehydrated, weight loss <20%, orbital tightening/eye discharge, irregular breathing, slightly reduced mobility, slightly reduced activity, sides pinched in, slight drop in temperature: animal can continue on study but should be monitored closely; 4 = piloerection, moderate persistent hunching for up to 1 h, dehydrated, weight loss >20%, orbital tightening/eye discharge, reduced breathing rate, increased breathing depth, subdued, reduced activity, cold to touch, pale in color: animal has reached moderate endpoint and should be euthanized.

#### Inoculations

To prepare cell suspension for the inoculations, *M. pneumoniae* strains were grown in a T75 culture flask (Sarstedt) with Hayflick-ampicillin 100 µg ml^−1^ (H-Amp_100_) broth (37 °C, 3–4 d, 5% CO_2_). After washing, cells were scraped with PBS and passed through a syringe (25G). Appropriate suspensions (c.f.u. µl^−1^ indicated in each experiment) were prepared and an aliquot was plated on H-Amp_100_ agar to assess bacterial counts. *P. aeruginosa* PAO1 was grown overnight in TSB broth at 37 °C on shaking and diluted with PBS to an optimal concentration. For the efficacy studies, mice were rendered neutropenic with two subcutaneous injections of cyclophosphamide: 150 mg kg^–1^ at 4 d before PAO1 infection and 100 mg kg^–1^ at 1 d before PAO1 infection. Before inoculation with *M. pneumoniae* or PAO1, mice were anesthetized with isoflurane 2% (ISOFLO, Covegan). Intratracheal inoculation was performed by introducing 100 µl of cell suspension through a sterile 20G (1.1-mm diameter) Vialon intravenous catheter (Becton-Dickinson) inserted into the trachea. Intranasal inoculation was performed by pipetting 20 µl of cell suspension into both nostrils (total volume was 40 µl). Mice were then released into their cages and monitored until they regained consciousness.

#### Mouse sampling

Mice were necropsied at 2, 14 and 45 d.p.i. Lungs were collected and processed for histological studies according to Morton and Snider^65^. Briefly, lungs were insufflated with 10% formalin through the trachea in situ and then removed and fixed in formalin for 24 h for histological analysis.

For RNA extraction, lungs were sectioned and immediately frozen in liquid nitrogen and stored at –80 °C until use. When required, BALF samples were obtained by intratracheal perfusion and harvesting of 0.7 ml of PBS pe rmouse, with a sterile 20G Vialon catheter.

For bacteriological analysis, lung and BALF samples were serially tenfold diluted in sterile PBS and plated by triplicate to determine the number of viable bacteria on Hayflick-ampicillin agar (for *M. pneumoniae*) or *Pseudomonas* agar (Oxoid, for *P. aeruginosa*). Plates were incubated at 37 °C for 14 d (for *M. pneumoniae*) or 16–24 h (for *P. aeruginosa*), and then colonies were counted using a Leica Zoom 2000 plate microscope at ×10 magnification.

### Lung RNA extraction and RT–qPCR analysis

Lungs previously stored at –80 °C were homogenized using Ultra-Turrax (IKA) and total RNA was isolated using RNeasy Mini Kit (QIAGEN), following the manufacturer’s instructions. RNA concentrations were measured spectrophotometrically using Nanodrop One (Thermo Fisher Scientific), and sample RNA integrity was confirmed by 1% agarose gel electrophoresis. RNA samples with the ratio of absorbance at 260 nm:280 nm of 1.8–2.1 were used. Complementary DNA from whole lung cells was synthesized from total RNA (1 μg) using SuperScript II Reverse Transcriptase reagents (Invitrogen). PCR amplification was performed using SYBR Premix Ex Taq II (Tli RNaseH Plus; Takara) and fluorescence was analyzed with AriaMx Real-Time PCR System (Agilent Technologies). The comparative threshold cycle (*C*_*t*_) method^70^ was used to obtain relative quantities of messenger RNAs that were normalized using *gapdh* as an endogenous control. Primer sequences for the genes *tnf*, *il1b*, *il6*, *il12a*, *il23a*, *ifng*, *ccl2*, *ccl3*, *cxcl1* and *gapdh* are shown in Supplementary Table 7.

### Histopathological analysis of lung samples

After necropsy, lung samples were insufflated with formalin, fixed for at least 24 h, trimmed and then automatically processed in ethanol series and xylene substitute (Citadel 2000 Tissue Processor, Thermo Fisher Scientific). Thereafter, tissues were embedded in paraffin (HistoStar Embedding Workstation, Thermo Fisher Scientific), sectioned at 4 μm (Finesse ME + Microtome, Thermo Fisher Scientific), stained with haematoxylin and eosin (H&E) (Gemini AS Automated Slide Stainer, Thermo Fisher Scientific) and mounted on glass slides (CTM6 Coverslipper Thermo Fisher Scientific). For light microscopy analysis, histological images were obtained using a digital camera (MC170 HD, Leica) connected to an optical microscope (DM2000, Leica) using a commercial software (Leica Application Suite, v.4.6.0). Sections were examined blind as sets by a trained veterinary pathologist and lesions were scored based on five parameters: (A) peribronchial/peribronchiolar inflammatory infiltrate affectation (0, none; 1, <25%; 2, 25–75%, 3, >75%); (B) peribronchial/peribronchiolar inflammatory infiltrate intensity (0, none; 1, incomplete infiltration; 2, complete infiltration, <5 cells thick; 3, complete infiltration, ≥5 cells thick); (C) bronchial/bronchiolar luminal exudate intensity (0, none; 1, <25% of lumen occlusion; 2, >25% of lumen occlusion); (D) perivascular inflammatory infiltrate affectation (0, none; 1, <10%; 2, 10–50%; 3, >50%); and (E) interstitial pneumonia intensity (0, none; 3, multifocal foci of interstitial pneumonia; 5, multifocal-coalescing foci to diffuse interstitial pneumonia). According to previous studies^62,63^, a final score (0–26) was obtained with the formula A + (3 × (B + C)) + D + E.

### Dispersal of ETT biofilms from patients

ETTs from patients receiving MV were collected from September 2015 to December 2017. They were selected from patients with a positive respiratory culture for *P. aeruginosa* during intubation, ventilated over 11 (6.00–16.50) d and in the confirmed presence of *P. aeruginosa* in ETTs (2.57 (2.20–4.81) log_10_(c.f.u. ml^–1^)) at extubation of the patient. ETTs were frozen until analysis, in a collection carried out in compliance with the Declaration of Helsinki (current version, Fortaleza, Brazil, October 2013). The collection was approved by the institution’s internal review board (Ethical Committee for Research in Medicines, Hospital Clinic of Barcelona, Spain), with the registry no. R190311-203;HCB/2019/026. Afterwards, ETTs were slowly unfrozen to room temperature and sliced into the following sections with different treatments: control (Hayflick medium alone without treatment); CV2_HA_PL1 (~1 × 10^8^ log(c.f.u. ml^–1^)), C/T (Zerbaxa 1 g per 0.5 g powder, 5 µg ml^–1^) and CV2_HA_P1 + C/T. The CV2_HA_P1 inoculum contained 7.32 (8.26–9.20) log(c.f.u. ml^–1^) (Fig. 4a)^71^.

Each ETT section and treatment condition were adjusted to a final volume of 1,600 µl with Hayflick medium and incubated at 37 °C for 24 h. Before diluting (–1 to –5(log_10_)) and culturing on MacConkey and blood agar (Becton-Dickinson GmbH), samples were sonicated for 5 min at 40 kHz in ultrasound-cleaning equipment (Branson 3510 E-MT; Bransonic) as previously published^71^. *P. aeruginosa* counts in each treatment were reported in log_10_(c.f.u. ml^–1^). A complete antibiogram (amikacin, colistin, piperacillin/tazobactam, aztreonam, tobramycin, ceftazidime, meropenem or imipenem) was performed for each *P. aeruginosa* strain using the Kirby–Bauer method, with the *P. aeruginosa* American Type Culture Collection 27853 strain as a control. The interpretation of results was carried out according to the European Committee on Antimicrobial Susceptibility Testing. *P. aeruginosa* was considered to be multidrug resistant when nonsusceptible to three or more families of antipseudomonal antimicrobials^72^. In addition, molecular epidemiology was analyzed by multilocus sequence typing (https://pubmlst.org/paeruginosa). Phylogenetic analysis was carried out using the eBURST algorithm (http://www.phyloviz.net/goeburst).

### Statistical analysis

Categorical variables were reported as number (%), whereas continuous variables were reported as mean ± s.d. or median (interquartile range (IQR)), if the distribution was normal or non-normal, respectively. Continuous variables between groups were compared using the one-way analysis of variance (ANOVA) followed by a post-hoc, pairwise Tukey’s honestly significant difference (HSD) or a Kruskal–Wallis test, as appropriate. Paired samples were compared using the paired Student’s *t*-test or nonparametric Wilcoxon’s signed-rank test when appropriate. Spearman’s correlation analyses were performed to determine associations between continuous variables. In all the cases, *P* ≤ 0.05 was considered to be statistically significant and the exact *P* value is specified in figure legends when possible. Data were analyzed using StatsDirect software (v.3.1.8) or GraphPad Prism (v.8.0.1).

### Reporting summary

Further information on research design is available in the Nature Portfolio Reporting Summary linked to this article.

## Online content

Any methods, additional references, Nature Portfolio reporting summaries, source data, extended data, supplementary information, acknowledgements, peer review information; details of author contributions and competing interests; and statements of data and code availability are available at 10.1038/s41587-022-01584-9.

## Supplementary information

Supplementary Information Supplementary Tables 1–7

Reporting Summary

## Data Availability

The MS dataset has been submitted to the public data repository PRIDE (PRoteomics IDEntifications database) with the following accession no.: PXD037233.
