# Supplementary material for: Engineered live bacteria suppress Pseudomonas aeruginosa infection in mouse lung and dissolve endotracheal-tube biofilms
Source: Nat Biotechnol. 2023 Jan 19;41(8):1089–98. doi: 10.1038/s41587-022-01584-9 (PMC10421741; doi:10.1038/s41587-022-01584-9)
Supplement: Supplementary file 1 — Supplementary Tables 1–7 [file 41587_2022_1584_MOESM1_ESM.pdf]

nature biotechnology

Article

https://doi.org/10.1038/s41587-022-01584-9

Engineered live bacteria suppress *Pseudomonas aeruginosa* infection in mouse lung and dissolve endotracheal-tube biofilms

In the format provided by the authors and unedited

SUPPLEMENTARY TABLES:

Supplementary Table 1. Histopathologic evaluation of the lung lobes (left, right cranial and right medium) of mice inoculated with 1 x 10^7 CFU of the WT or CV2 *M. pneumoniae* strains or with PBS at 2, 14 or 45 days post-infection (dpi). Data represent the average and the SD of the evaluations from five mice per group. The scoring system is detailed in the Methods section.

			Peribronchiolar/bronchial infiltrate (%)			Quality of peribronchiolar/bronchial infiltrates			Bronchiolar/bronchial luminal exudate			Perivascular infiltrate (%)			Parenchymal pneumonia			Total score (0-26)		
Time post-infection	Group		Left	RCr	RM	Left	RCr	RM	Left	RCr	RM	Left	RCr	RM	Left	RCr	RM	Left	RCr	RM
2 dpi	PBS	Average	0.0	0.0	0.0	0.0	0.0	0.0	0.0	0.0	0.0	0.2	0.2	0.0	0.0	0.0	0.0	0.2	0.2	0.0
		SD	0.0	0.0	0.0	0.0	0.0	0.0	0.0	0.0	0.0	0.4	0.4	0.0	0.0	0.0	0.0	0.4	0.4	0.0
	WT	Average	0.8	0.4	1.0	0.8	0.4	1.0	2.0	1.2	1.6	1.8	1.8	1.6	2.2	2.8	3.0	13.2	9.8	13.4
		SD	0.4	0.5	0.0	0.4	0.5	0.0	0.0	1.0	0.8	0.4	0.4	0.5	1.9	1.6	0.0	2.1	4.5	2.7
	CV2	Average	0.8	0.2	0.4	0.8	0.2	0.4	0.8	0.8	0.4	1.6	1.0	1.4	0.6	1.2	0.0	7.8	5.4	4.2
		SD	0.4	0.4	0.5	0.4	0.4	0.5	1.0	1.0	0.8	0.5	0.0	0.5	1.2	1.5	0.0	4.0	3.0	3.9
14 dpi	PBS	Average	0.0	0.0	0.0	0.0	0.0	0.0	0.0	0.0	0.0	0.2	0.2	0.0	0.0	0.0	0.0	0.2	0.2	0.0
		SD	0.0	0.0	0.0	0.0	0.0	0.0	0.0	0.0	0.0	0.4	0.4	0.0	0.0	0.0	0.0	0.4	0.4	0.0
	WT	Average	0.8	0.4	1.0	0.8	0.8	0.8	2.0	1.2	1.6	1.8	1.8	1.6	2.2	2.8	3.0	13.2	9.8	13.4
		SD	0.4	0.5	0.0	0.4	0.4	0.4	0.0	1.0	0.8	0.4	0.4	0.5	1.9	1.6	0.0	2.1	4.5	2.7
	CV2	Average	0.8	0.2	0.4	0.0	0.0	0.0	0.8	0.8	0.4	1.6	1.0	1.4	0.6	1.2	0.0	7.8	5.4	4.2
		SD	0.4	0.4	0.5	0.0	0.0	0.0	1.0	1.0	0.8	0.5	0.0	0.5	1.2	1.5	0.0	4.0	3.0	3.9
45 dpi	PBS	Average	0.0	0.0	0.0	0.0	0.0	0.0	0.0	0.0	0.0	0.2	0.2	0.0	0.0	0.0	0.0	0.2	0.2	0.0
		SD	0.0	0.0	0.0	0.0	0.0	0.0	0.0	0.0	0.0	0.4	0.4	0.0	0.0	0.0	0.0	0.4	0.4	0.0
	WT	Average	0.8	0.4	1.0	0.0	0.0	0.0	2.0	1.2	1.6	1.8	1.8	1.6	2.2	2.8	3.0	13.2	9.8	13.4
		SD	0.4	0.5	0.0	0.0	0.0	0.0	0.0	1.0	0.8	0.4	0.4	0.5	1.9	1.6	0.0	2.1	4.5	2.7
	CV2	Average	0.8	0.2	0.4	0.0	0.0	0.0	0.8	0.8	0.4	1.6	1.0	1.4	0.6	1.2	0.0	7.8	5.4	4.2
		SD	0.4	0.4	0.5	0.0	0.0	0.0	1.0	1.0	0.8	0.5	0.0	0.5	1.2	1.5	0.0	4.0	3.0	3.9

Supplementary Table 2. Gene expression of cytokines analyzed in lungs of mice inoculated with WT or CV2 M. pneumoniae or with PBS. Analysis was performed in three major lung lobes (RCr, right cranial; RM, right middle; and L, left). Values are presented as mean ± SD; n = 5 mice per group.

Gene	Group	2 dpi			14 dpi			45 dpi		
		RCr	RM	L	RCr	RM	L	RCr	RM	L
tnf	PBS	1 ± 1.2	1 ± 0.4	1 ± 0.9	1 ± 1.0	1 ± 1.0	1 ± 0.8	1 ± 0.7	1 ± 0.8	1 ±0.0
	WT	2.8 ± 2.4	3.1 ± 2.4	3.3 ± 0.2	3.4 ± 2.5	1.9 ± 0.2	2.2 ± 0.0	1.4 ± 0.5	2.11 ± 1.6	2.2 ± 1.4
	CV2	1.1 ± 0.3	1.8 ± 0.6	1.5 ± 0.8	0.5 ± 0.4	0.4 ± 0.7	0.4 ± 0.1	1.2 ± 0.4	1.7 ± 0.3	1.9 ± 0.3
cxcl1	PBS	1 ± 0.3	1 ± 0.3	1 ± 0.9	1 ± 0.5	1 ± 0.4	1 ± 0.6	1 ± 0.2	1 ± 0.3	1 ± 0.8
	WT	3.8 ± 1.6	3.5 ± 1.8	4.4 ± 0.1	0.8 ± 0.6	0.7 ± 0.2	0.1 ±0.0	1.2 ± 0.6	1.2 ± 0.8	1.9 ± 0.0
	CV2	1.3 ± 0.7	0.94 ± 0.4	1.3 ± 0.7	0.4 ± 0.4	0.3 ± 0.1	0.2 ± 0.1	0.7 ± 0.3	0.7 ± 0.6	0.9 ± 0.2
ccl3	PBS	1 ± 0.3	1 ± 0.5	1 ± 0.6	1 ± 0.4	1 ± 0.7	1 ± 0.5	1 ± 0.6	1 ± 0.7	1 ± 0.5
	WT	4.0 ± 0.7	3.2 ± 2.4	4.4 ±0.1	2.7 ± 1.3	1.6 ± 0.4	1 ± 0.0	0.7 ± 0.7	1.5 ± 0.8	1 ± 0.5
	CV2	1.9 ± 0.1	1.2 ± 0.5	2.2 ± 0.8	1.5 ± 0.6	1.5 ± 1.4	1.7 ± 0.4	0.8 ± 0.5	1.0 ± 0.4	1.6 ± 0.2
ccl2	PBS	1 ± 0.3	1 ± 0.2	1 ± 0.5	1 ± 0.5	1 ± 0.4	1 ± 0.5	1 ± 0.4	1 ± 0.3	1 ± 0.0
	WT	3.8 ± 0.7	2.8 ± 1.1	2.4 ± 0.3	2.4 ± 1.3	1.9 ± 0.5	2.2 ± 0.0	2.3 ± 3.4	1.3 ± 0.4	1.5 ± 0.9
	CV2	1.4 ± 0.3	0.8 ± 0.2	0.9 ± 0.6	1.1 ± 0.2	1.3 ± 0.7	1.8 ± 0.3	0.8 ± 0.5	0.6 ± 1.1	1.3 ± 0.6
ifng	PBS	1 ± 0.3	1 ± 0.6	1 ± 0.6	1 ± 0.6	1 ± 0.6	1 ± 0.1	1 ± 0.1	1 ± 0.6	1 ± 0.5
	WT	4.5 ± 2.2	5.8 ± 3.4	5.7 ± 3.0	2.6 ± 1.4	1.1 ± 0.9	0.6 ± 0.0	1.7 ± 0.5	2.54 ± 1.2	1.8 ± 0.6
	CV2	1.0 ± 0.5	0.5 ± 0.1	1.3 ± 0.6	1.1 ± 0.7	0.9 ± 0.6	0.7 ± 0.1	2.0 ± 0.7	1.45 ± 1.7	1.7± 1.7
il1b	PBS	1 ± 0.5	1 ± 0.3	1 ± 0.7	1 ± 0.4	1 ± 0.4	1 ± 0.3	1 ± 0.3	1 ± 0.2	1 ± 0.2
	WT	3.3 ± 1.5	2.8 ± 1.5	2.9 ± 1.7	1.4 ± 0.1	1.0 ± 0.4	0.9 ± 0.0	0.9 ± 0.2	1.0 ± 0.4	1.3 ± 0.1
	CV2	1.6 ± 0.7	1.2 ± 0.1	1.4 ± 0.9	1.1 ± 0.8	0.6 ± 0.1	1.1 ± 0.4	0.7 ± 0.3	0.5 ± 0.5	1.4 ± 0.9
il6	PBS	1 ± 0.3	1 ± 0.1	1 ± 0.8	1 ± 0.3	1 ± 0.6	1 ± 0.8	1 ± 0.8	1 ± 0.0	1 ± 0.1
	WT	0.7 ± 0.5	0.4 ± 0.2	0.5 ± 0.2	2.3 ± 0.7	2.1 ± 0.2	1.9 ± 0.0	1.5 ± 0.3	1.1 ± 0.2	0.8 ± 0.0
	CV2	0.4 ± 0.1	0.8 ± 0.5	0.7 ± 0.3	0.9 ± 0.3	0.9 ±0.4	1.2 ±0.2	0.7 ± 0.2	0.5 ± 0.4	1.1 ± 0.5
il12a	PBS	1 ± 0.3	1 ± 0.3	1 ± 0.5	1 ± 0.5	1 ± 0.5	1 ± 0.3	1 ± 0.3	1 ± 0.4	1 ± 0.1
	WT	3.3 ± 1.5	4.2 ± 1.6	2.7 ± 1.6	2.1 ± 0.8	0.9 ± 0.4	1.2 ± 0.0	1.4 ± 0.9	1.7 ± 0.9	2.2 ± 1.2
	CV2	1.7 ± 1.0	1.7 ± 0.9	1.5 ± 1.2	1.3 ± 0.6	0.5 ± 0.2	1.6 ± 0.4	1.6 ± 1.2	1.0 ± 0.7	1.5 ± 0.7
il23a	PBS	1 ± 0.6	1 ± 0.3	1 ± 0.5	1 ± 0.5	1 ± 0.5	1 ± 0.3	1 ± 0.4	1 ± 0.4	1 ± 0.5
	WT	1.3 ± 0.2	1.0 ± 0.1	0.9 ± 0.5	0.7 ± 0.4	0.9 ± 0.5	0.3 ± 0.0	1.0 ± 2.2	0.9 ± 1.2	1.0 ± 0.4
	CV2	1.1 ± 0.6	1.2 ± 0.5	0.5 ± 0.3	0.8 ± 0.6	1.0 ± 0.7	0.9 ± 0.6	0.7 ± 0.4	0.6 ± 0.3	1 ± 0.6

Supplementary Table 3. Description of different vectors used to obtain the different engineered strains.

Vector name	Vector description	Transformed strain	Obtained strain name
pMTnCmLox_EfTu_MPN142(OPT)_AI-II´	Minitransposon vector used to obtain strains that express and secrete AI-II'	WT and CV2	WT_A; CV2_A
pMTnTclox_EfTu_MPN142(OPT)_PelAh_Flag	Minitransposon vector used to obtain strains that express and secrete PelAh	WT and CV2	WT_PelAh; CV2_PelAh
pMTnTclox_Eftu_MPN142(OPT)_PslGh_Flag	Minitransposon vector used to obtain strains that express and secrete PslGh	WT and CV2	WT_PslGh; CV2_PslGh
pMTnTclox_EfTu_MPN142(OPT)_PelAh_ Flag_Eftu_MPN142(OPT)_PslGh_Flag	Minitransposon vector used to obtain strains that express and secrete PelAh and PslGh	WT and CV2; WT_A and CV2_A	WT_H; CV2_H; WT_HA; CV2_HA
pMTnGmLox_P3_MPN142(OPT2)_pyocin_L1	Minitransposon vector used to express and secrete of PyocinL1 without Flag	WT; CV2 WT_HA and CV2_HA	WT_PL1; CV2_PL1; WT_HA_PL1 and CV2_HA_PL1
pMTnGmLox_P3_MPN142(OPT2)_pyocin_S5	Vector to test secretion of PyocinS5 without Flag. Expression of gene under P3 promoter	WT	WT_PS5

Supplementary Table 4. Average values of areas under curve (AUC) for the three best peptides obtained by MS for the heterologous proteins identified in cells and supernatants (SPN) of different *M. pneumoniae* strains. (ND, Not Detected).

Protein	CV2_HA (Cells)	CV2_HA (SPN)	CV2_A1 -II' (Cells)	CV2_A1-II' (SPN)	CV2_ PelAh-PslGh (Cells)	CV2_ PelAh-PslGh (SPN)	CV2_HA_ P1 (Cells)	CV2_HA_ P1 (SPN)
AI-II'	8,27E+07	9.87E+07	5.87E+0 7	3.83E+07	ND	ND	6.97E+07	2.17E+08
PelAh	1.43E+08	2.40E+07	ND	ND	1.43E+08	8.53E+07	1.40E+08	6.30E+07
PslGh	8.87E+08	4.10E+07	ND	ND	8.27E+08	9.97E+07	9.00E+08	6.40E+07
Pyocin-L1	ND	ND	ND	ND	ND	ND	7.27E+08	7.85E+07

Supplementary Table 5. Histopathologic evaluation of the lung lobes (left, right cranial and right medium) of mice inoculated with 1 x 10^8 CFU of the WT or CV2_HA_P1 *M. pneumoniae* strains or with PBS at 2-, 14- and 45 days post-infection (dpi). The indicated numbers represent the average and the SD of the evaluations from five mice per group. The scoring system is detailed in the Methods section.

			Peribronchial/bronchial infiltrate (%)			Quality of peribronchial/bronchial infiltrates			Bronchiolar/bronchial luminal exudate			Perivascular infiltrate (%)			Parenchymal pneumonia			Total score (0-26)		
Time post-infection	Group		Left	RCr	RM	Left	RCr	RM	Left	RCr	RM	Left	RCr	RM	Left	RCr	RM	Left	RCr	RM
2 dpi	PBS	Mean	0.0	0.0	0.0	0.0	0.0	0.0	0.0	0.0	0.0	0.2	0.2	0.0	0.0	0.0	0.0	0.2	0.2	0.0
		SD	0.0	0.0	0.0	0.0	0.0	0.0	0.0	0.0	0.0	0.4	0.4	0.0	0.0	0.0	0.0	0.4	0.4	0.0
	WT	Mean	1.4	1.6	1.4	1.4	1.6	1.4	2.0	1.8	1.2	2.0	1.4	1.4	3.2	2.8	1.6	16.8	16.0	12.2
		SD	0.5	0.5	0.5	0.8	0.5	0.5	0.0	0.4	0.4	0.6	0.5	0.5	1.8	1.6	2.1	4.2	4.2	4.7
	CV2_HA_P1	Mean	0.4	0.6	0.2	0.4	0.6	0.2	1.6	1.4	1.6	1.6	1.8	2.0	0.6	1.2	0.6	8.6	9.6	8.2
		SD	0.5	0.5	0.4	0.5	0.5	0.4	0.5	0.5	0.5	0.8	0.4	0.6	1.2	1.5	1.2	3.2	1.6	4.2
14 dpi	PBS	Mean	0.0	0.0	0.0	0.0	0.0	0.0	0.0	0.0	0.0	0.2	0.2	0.0	0.0	0.0	0.0	0.2	0.2	0.0
		SD	0.0	0.0	0.0	0.0	0.0	0.0	0.0	0.0	0.0	0.4	0.4	0.0	0.0	0.0	0.0	0.4	0.4	0.0
	WT	Mean	0.2	0.4	0.2	0.4	0.4	0.2	0.8	0.6	0.4	1.6	2.0	1.6	1.2	0.6	0.6	6.6	6.0	4.2
		SD	0.4	0.5	0.4	0.8	0.5	0.4	0.4	0.5	0.5	0.5	0.0	0.5	1.5	1.2	1.2	1.7	2.7	2.8
	CV2_HA_P1	Mean	0.0	0.0	0.0	0.0	0.0	0.0	0.0	0.0	0.0	1.4	1.0	1.2	1.2	0.6	0.6	2.6	1.6	1.8
		SD	0.0	0.0	0.0	0.0	0.0	0.0	0.0	0.0	0.0	0.5	0.6	0.4	1.5	1.2	1.2	1.6	1.7	1.2
45 dpi	PBS	Mean	0.0	0.0	0.0	0.0	0.0	0.0	0.0	0.0	0.0	0.2	0.2	0.0	0.0	0.0	0.0	0.2	0.2	0.0
		SD	0.0	0.0	0.0	0.0	0.0	0.0	0.0	0.0	0.0	0.4	0.4	0.0	0.0	0.0	0.0	0.4	0.4	0.0
	WT	Mean	0.0	0.0	0.0	0.0	0.0	0.0	0.0	0.0	0.0	1.0	1.0	1.0	0.6	1.2	0.6	1.6	2.2	1.6
		SD	0.0	0.0	0.0	0.0	0.0	0.0	0.0	0.0	0.0	0.0	0.9	0.0	1.2	1.5	1.2	1.2	2.3	1.2
	CV2_HA_P1	Mean	0.0	0.0	0.0	0.0	0.0	0.0	0.0	0.0	0.0	0.2	0.2	0.2	0.0	0.0	0.0	0.2	0.2	0.2
		SD	0.0	0.0	0.0	0.0	0.0	0.0	0.0	0.0	0.0	0.4	0.4	0.4	0.0	0.0	0.0	0.4	0.4	0.4

Supplementary Table 6. Gene expression of cytokines analyzed in lungs of mice inoculated with 1 x 10^8 CFU of WT or CV2_HA_P1 M. pneumoniae or with PBS. Analysis was performed on the left lung and values are presented as mean ± SD (n = 5 mice per group).

Gene	Group	2 dpi	14 dpi
tnf	PBS	1.0 ± 0.5	1.0 ± 0.5
	WT	18.0 ± 1.8	3.0 ± 0.7
	CV2_HA_P1	5.1 ± 2.2	9.5 ± 3.5
cxcl1	PBS	1.0 ± 0.5	1.0 ± 0.5
	WT	715.4 ± 246.0	3.4 ± 1.5
	CV2_HA_P1	343.3 ± 281.9	1.25 ± 0.3
ccl3	PBS	1.0± 0.67	9.4 ± 3.5
	WT	85.4 ± 62.9	3.0 ± 0.7
	CV2_HA_P1	31.6 ± 32.6	1.0 ± 0.5
ccl2	PBS	1.0 ± 0.5	1.0 ± 0.5
	WT	145.4 ± 18.4	3.4 ± 1.5
	CV2_HA_P1	115.2 ± 42.0	1.3 ± 0.3
Ifng	PBS	1.0 ± 0.3	1.0 ± 0.8
	WT	55.8 ± 7.0	0.9 ± 0.2
	CV2_HA_P1	26.6 ± 17.7	0.7 ± 0.1
il1b	PBS	1.0 ± 0.44	1.0 ± 0.4
	WT	40.3 ± 1.9	2.9 ± 1.0
	CV2_HA_P1	12.5 ± 1.4	1.7 ± 0.9
il6	PBS	1.0 ± 0.5	1.0 ± 0.9
	WT	65.9 ± 18.9	0.5 ± 0.1
	CV2_HA_P1	26.4 ± 22.7	0.4 ± 0.1
il12a	PBS	1.0 ± 0.5	1.0 ± 0.7
	WT	3.1 ± 0.9	1.5 ± 0.5
	CV2_HA_P1	2.1 ± 0.9	1.2 ± 0.4
il23a	PBS	1.0 ± 0.5	1.0 ± 0.5
	WT	13.4 ± 2.9	2.1 ± 0.3
	CV2_HA_P1	5.7 ± 0.9	1.4 ± 0.2

Supplementary Table 7. Oligonucleotides used in the qPCR to analyze the inflammation markers in lungs.

Gene	Forward (F)/reverse (R)	Sequence (5'-3')
gapdH	F	CCCACTAACATCAAATGGGG
	R	CCTTCCACAATGCCAAAGTT
tnf	F	AGGCACTCCCCCAAAAGA
	R	GCTCCTCCACTTGGTGGTTT
cxcl1	F	GACAG ACTGCTCTGATGGCA
	R	TGCACTT CTTTTCGCACAAC
ccl3	F	CCCAGCCAGGTGTCATTTTCC
	R	GCATTCAGTTCCAGGTCAGTG
ccl2	F	CTTCTGGGCCTGCTGTTCA
	R	CCAGCCTACTCATTGGGATCA
ifn	F	CTGGCAAAAGGATGGTGAC
	R	GCTGATGGCCTGATTGTCTT
il1b	F	GCTGCTTCCAAACCTTTGAC
	R	AGCTTCTCCACAGCCACAAT
il6	F	TGATGCACTTGCAGAAAACA
	R	GGTCTTGGTCCTTAGCCACTC
il12a	F	CAGCACCAGCTTCTTCATCAG
	R	TCTTCAAAGGCTTCATCTGCAA
il23a	F	*TGCTGGATTGCAGAGCAGTAA*
	R	GCATGCAGAGATTCCGAGAGA
