# Supplementary material for: Engineered live bacteria suppress Pseudomonas aeruginosa infection in mouse lung and dissolve endotracheal-tube biofilms
Source: Nat Biotechnol. 2023 Jan 19;41(8):1089–98. doi: 10.1038/s41587-022-01584-9 (PMC10421741; doi:10.1038/s41587-022-01584-9)
Supplement: Supplementary file 2 — Reporting Summary [file 41587_2022_1584_MOESM2_ESM.pdf]

nature research

Corresponding author(s): Maria Lluch-Senar & Luís Serrano

Last updated by author(s): Sep 30, 2022

Reporting Summary

Nature Research wishes to improve the reproducibility of the work that we publish. This form provides structure for consistency and transparency in reporting. For further information on Nature Research policies, see our Editorial Policies and the Editorial Policy Checklist.

Statistics

For all statistical analyses, confirm that the following items are present in the figure legend, table legend, main text, or Methods section.

n/a	Confirmed	
☐	☒	The exact sample size (n) for each experimental group/condition, given as a discrete number and unit of measurement
☐	☒	A statement on whether measurements were taken from distinct samples or whether the same sample was measured repeatedly
☐	☒	The statistical test(s) used AND whether they are one- or two-sided<br>*Only common tests should be described solely by name; describe more complex techniques in the Methods section.*
☐	☒	A description of all covariates tested
☐	☒	A description of any assumptions or corrections, such as tests of normality and adjustment for multiple comparisons
☐	☒	A full description of the statistical parameters including central tendency (e.g. means) or other basic estimates (e.g. regression coefficient) AND variation (e.g. standard deviation) or associated estimates of uncertainty (e.g. confidence intervals)
☐	☒	For null hypothesis testing, the test statistic (e.g. F, t, r) with confidence intervals, effect sizes, degrees of freedom and P value noted<br>*Give P values as exact values whenever suitable.*
☒	☐	For Bayesian analysis, information on the choice of priors and Markov chain Monte Carlo settings
☐	☒	For hierarchical and complex designs, identification of the appropriate level for tests and full reporting of outcomes
☐	☒	Estimates of effect sizes (e.g. Cohen's d, Pearson's r), indicating how they were calculated

Our web collection on statistics for biologists contains articles on many of the points above.

Software and code

Policy information about availability of computer code

Data collection	No software was used
Data analysis	goeBURST algorithm, G*Power software (3.1), StatsDirect software (3.1.8) or GraphPad Prism (8.0.1)

For manuscripts utilizing custom algorithms or software that are central to the research but not yet described in published literature, software must be made available to editors and reviewers. We strongly encourage code deposition in a community repository (e.g. GitHub). See the Nature Research guidelines for submitting code & software for further information.

Data

Policy information about availability of data

All manuscripts must include a data availability statement. This statement should provide the following information, where applicable:

- Accession codes, unique identifiers, or web links for publicly available datasets
- A list of figures that have associated raw data
- A description of any restrictions on data availability

The authors declare that the data supporting the findings of this study are available within the paper.

Field-specific reporting

Please select the one below that is the best fit for your research. If you are not sure, read the appropriate sections before making your selection.

☒ Life sciences ☐ Behavioural & social sciences ☐ Ecological, evolutionary & environmental sciences

For a reference copy of the document with all sections, see nature.com/documents/nr-reporting-summary-flat.pdf

Life sciences study design

All studies must disclose on these points even when the disclosure is negative.

Sample size	Sample size was calculated using G*power software with the following parameter: error α:0.05; potency 1-β :0.8, values for parameter "final score of pulmonary lesions" based in previous experiment (average ±SD) 12.1±6 y 5.8±2.5, obtaining as a output n=5 mice per group.
Data exclusions	In the ex-vivo assays with sections of endotracheal tubes, one control sample was discarded because no P. aeruginosa counts could be obtained due to overgrowth of Proteus species.
Replication	All the in vitro assays were carried out using al least triplicate samples; all the assays were reproduced at least three independent times. In vivo assays with mice were performed with at least 6 animals in each experimental group, and all attempts at replication were successful. For any other epxeriments, independent experiments with triplicate samples were always performed.
Randomization	All the mice in the in vivo are commercial inbred strains assay, carrying the same genetic background. Hence, the animals were allocated randomly in each group. In the assays with ex-vivo endotracheal tubes, section of each tube were allocated randomly into experimental groups.
Blinding	The analysis of the ex-vivo samples (i.e. histopathology analysis) was analysed by a pathologist that did not know the ID of the samples. For experiments other than histopathology analysis, the investigators were also blinded to group allocation during data collection and/or analysis (i.e. animal health scoring during survival curves, counting of bacterial CFUs on plates). No blinding was required when objective data (i.e. absorbance values provided by plate reader, animals weights, Ct values from RT-qPCR) were analysed and plotted on graphs.

Reporting for specific materials, systems and methods

We require information from authors about some types of materials, experimental systems and methods used in many studies. Here, indicate whether each material, system or method listed is relevant to your study. If you are not sure if a list item applies to your research, read the appropriate section before selecting a response.

Materials & experimental systems

n/a	Involved in the study	
☒	☐	Antibodies
☒	☐	Eukaryotic cell lines
☒	☐	Palaeontology and archaeology
☐	☒	Animals and other organisms
☐	☒	Human research participants
☒	☐	Clinical data
☒	☐	Dual use research of concern

Methods

n/a	Involved in the study	
☒	☐	ChIP-seq
☒	☐	Flow cytometry
☒	☐	MRI-based neuroimaging

Animals and other organisms

Policy information about studies involving animals; ARRIVE guidelines recommended for reporting animal research

Laboratory animals	CD1 mice (female and male, 18–22 g, aged 4-6 weeks) were purchased from Charles River Laboratories (France)
Wild animals	The study did not involve wild animals
Field-collected samples	The study did not involve samples collected from the fied
Ethics oversight	- In vivo experiments produced by University of Navarra (Spain): Animal handling and procedures were in accordance with the current European (Directive 86/609/EEC) and National (Real Decreto 53/2013) legislations, following the FELASA and ARRIVE guidelines and with the approval of the Universidad Pública de Navarra (UPNa) Animal Experimentation Committee (Comité de Ética, Experimentación Animal y Bioseguridad) and the local Government authorization.<br>- In vivo experiments produced by Evotec (UK): all in vivo animal studies were performed in the UK under the UK Home Office License PA67E0BAA, with the clearance of the local ethical committee Animal Welfare and Ethical Review Body.

Note that full information on the approval of the study protocol must also be provided in the manuscript.

Human research participants

Policy information about studies involving human research participants

Population characteristics	Endotracheal tubes were obtained from mechanically ventilated patients admitted to an Intensive Care Medicine Unit (Hospital Clinic of Barcelona) with ventilator associated pneumonia (VAP) due to P. aeruginosa
Recruitment	September 2015 to December 2017
Ethics oversight	Collection R190311-203; HCB/2019/0262. Approved by the Ethical Committee for Research in Medicines, Hospital Clinic of Barcelona, Spain.

Note that full information on the approval of the study protocol must also be provided in the manuscript.
